# Mechanism of cotranslational modification of histones H2A and H4 by MetAP1 and NatD

**DOI:** 10.1126/sciadv.aeb1017

**Published:** 2025-12-19

**Authors:** Denis Yudin, Mateusz Jaskolowski, Ziyi Fan, Nicolas Burg, Sowmya Chandrasekar, Alfred M. Lentzsch, Alain Scaiola, Adrian Bothe, Elke Deuerling, Martin Gamerdinger, Shu-ou Shan, Nenad Ban

**Affiliations:** ^1^ Department of Biology, Institute of Molecular Biology and Biophysics, ETH Zürich, Zürich, Switzerland.; ^2^ Division of Chemistry and Chemical Engineering, California Institute of Technology, Pasadena, CA, USA.; ^3^ Department of Biology, Molecular Microbiology, University of Konstanz, Konstanz, Germany.

## Abstract

The replication-dependent histones H2A and H4 are among the most highly expressed proteins in eukaryotes during the S phase to ensure packaging of replicated chromosomes. Nearly all newly synthesized H2A and H4 are N-terminally acetylated by N-terminal acetyltransferase D (NatD) following excision of the initiator methionine by methionine aminopeptidases (MetAPs). These modifications influence chromatin function, but how they occur cotranslationally on these exceptionally abundant and small proteins was not understood. Here, we show that the nascent polypeptide-associated complex controls the cotranslational modification of histones H2A and H4 by recruiting NatD and the upstream enzyme MetAP1 to ribosomes. MetAP1 and NatD cooperate on the ribosome to create a confined environment for the efficient sequential modification of the nascent histone chain. Our work provides a mechanistic model for the early steps of histone maturation.

## Teaser

NAC enables cotranslational N-terminal processing of histones H2A and H4 by recruiting MetAP1 and NatD at the ribosomal tunnel.

## INTRODUCTION

N-terminal acetylation (Nt-acetylation) is a ubiquitous irreversible modification of protein N termini found on approximately 50 to 80% of the eukaryotic proteome (*1*). The N-terminal acetyl group affects a variety of protein properties and functions including folding (*2*), aggregation propensity (*3*), half-life (*4*), localization (*5*), and assembly (*6*). Aberrant NAT activity is associated with defects in cellular and organismal physiology (*1*) and linked to a variety of diseases including developmental syndromes (*7*) and cancer (*8*).

Nt-acetylation is mediated by up to eight classes of N-terminal acetyltransferases (NATs A to H), which transfer an acetyl moiety from acetyl-coenzyme A (Ac-CoA) onto the α-amino group at the protein N terminus (*1*, *9*). NatA to NatE acetylate nascent proteins cotranslationally and differ in their substrate specificities: NatB, NatC, and NatE acetylate the initiator methionine (iMet), whereas NatA and NatD require iMet excision by methionine aminopeptidase 1 or 2 (MetAP1 and MetAP2, respectively) to acetylate the newly formed N terminus (*10*).

NatD is a single-subunit NAT that exclusively acetylates the N termini of replication-dependent histones H2A and H4 on the ribosome or in the nucleus (*11*–*14*). In higher eukaryotes, NatD recognizes the conserved N-terminal SGRG motif of H2A and H4, which becomes exposed following iMet removal (*13*, *14*). This sequential modification is highly efficient, with reported levels of Nt-acetylation ranging from 87 to 99% and depending on the phase of the cell cycle (*15*–*18*). By modifying the histone tails, NatD acts akin to an epigenetic writer enzyme and can affect chromatin structure and gene regulation (*19*, *20*). There is evidence of interplay between Nt-acetylation and other chromatin modifications: Both H4S1 phosphorylation and H4R3 methylation are down-regulated on N-terminally acetylated histone H4 (*21*–*23*). These modifications, in turn, impair Nt-acetylation by NatD and result in altered patterns of gene expression (*24*). NatD likely requires substantial amounts of cellular Ac-CoA to achieve high levels of H2A and H4 acetylation (*19*), which could also link its activity to the metabolic state of the cell: in budding yeast, caloric restriction leads to decreased H2A/H4 Nt-acetylation. Conversely, NatD depletion in murine and insect model systems increases lipid synthesis (*25*). Furthermore, NatD is linked to tumor growth and was found to be up-regulated in various cancers (*23*, *26*, *27*).

NATs are substoichiometric to ribosomes (*28*), raising questions about how limited amounts of NAT enzymes meet the modification demands of the eukaryotic proteome. This problem is especially acute for NatD. H2A and H4 are among the most abundant proteins in eukaryotic cells, and their levels nearly double during the S phase to package the newly replicated chromosomes (*29*). On the other hand, the level of NatD is 2% of that of ribosomes in HeLa cells (*28*). In addition, both H2A and H4 are small, approximately 100 amino acids, limiting the time window for NatD to complete their Nt-acetylation before translation is terminated. The exceptionally high expression level and short length of H2A and H4 therefore pose unique challenges for cotranslational processing, especially given the limited abundance of NatD. The mechanism that ensures the timely and coordinated iMet excision and Nt-acetylation of H2A and H4 on translating ribosomes is unknown.

Recent studies suggest that nascent protein modification enzymes rely on additional factors to increase their local concentrations at the ribosomal tunnel (*30*). In higher eukaryotes, the heterodimeric nascent polypeptide-associated complex (NAC), an abundant ribosome-bound regulator of protein biogenesis (*31*, *32*), recruits and activates NatA to assist in the Nt-acetylation of approximately 40% of the mammalian proteome (*33*, *34*). NAC also binds MetAP1, the enzyme acting upstream of NatA, to coordinate the sequential processing of newly synthesized proteins (*33*, *35*). In addition, NAC was identified as the key factor controlling the fidelity of protein targeting to the endoplasmic reticulum (ER) by the signal recognition particle (SRP) (*31*, *36*). Whether NAC also plays a role in ensuring that the majority of histones H2A and H4 are modified cotranslationally has not been studied.

In this study, we combine structural, biochemical, and cell biology approaches to demonstrate that NAC controls the cotranslational Nt-acetylation of H2A and H4 by NatD. Recruited by the flexibly tethered ubiquitin-associated (UBA) domain of the NACα subunit, NatD selectively docks at the ribosomal tunnel exit of H2A- and H4-translating ribosomes, where it cooperates with the upstream enzyme MetAP1, recruited by the NACβ subunit of NAC, to sequentially process nascent histone chains. This spatially confined environment enables efficient and highly coordinated processing of these exceptionally abundant and unusually short protein substrates, establishing a model for NAC-facilitated early histone maturation at the ribosome.

## RESULTS

### NAC enhances NatD binding and activity on ribosomes

**Fig. 1.**
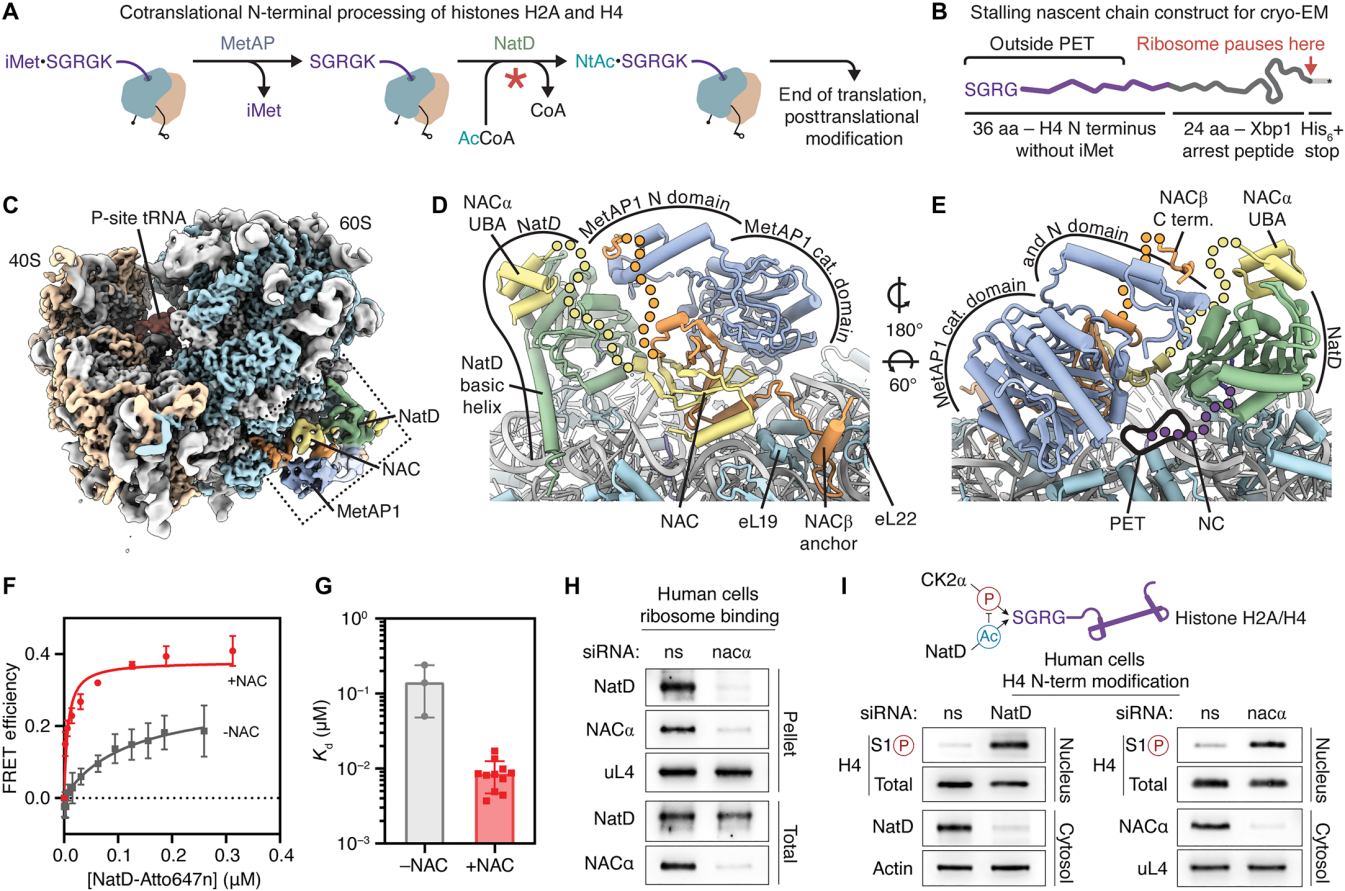
NAC recruits MetAP1 and NatD to translating ribosomes. (**A**) Schematic depicting cotranslational processing of H2A and H4 by MetAP and NatD. Red asterisk marks the reaction step at which we attempted to arrest the complex in the cryo-EM experiment. (**B**) Design of the nascent chain for the RNC used in the cryo-EM experiment. (**C**) Cryo-EM map of the RNC_H4-60aa_-NAC-MetAP1-NatD complex filtered to estimated local resolution, with rRNA in light gray, small subunit proteins in beige, large subunit proteins in light blue, P-site tRNA in red, NACα in yellow, NACβ in orange, MetAP1 in slate blue, and NatD in green. The semitransparent map segment corresponding to the N domain of MetAP1 was extracted from the map filtered to 10 Å resolution. (**D** and **E**) Details of the RNC_H4-60aa_-NAC-MetAP1-NatD model showing NAC, MetAP1, and NatD bound over the polypeptide exit tunnel (PET). Flexible segments of proteins not resolved in the structure are shown as dotted lines; the nascent chain (NC) is shown in purple. (**F** and **G**) Equilibrium titrations to measure the binding affinity of NatD for RNC_H4-70aa_ in the presence and absence of NAC (F). The lines are fits of the data to Eq. 2, and the obtained *K*_d_ values are summarized in (G). Values in (G) are shown as mean ± SD (*n* = 3 for the −NAC sample and *n* = 10 for the +NAC sample), with the dots showing the *K*_d_ from independent titrations. (**H**) Ribosome association of NatD after knockdown of NACα in HEK293T cells. Proteins in total and ribosomal pellet fractions were detected by immunoblotting. (**I**) Analysis of the *N*-acetyl-antagonistic histone phosphorylation mark at serine 1 (S1P) of H4 [mediated by Casein kinase 2α (CK2α) (*23*)] after knockdown of NatD (left) and NACα (right) in HEK293T cells. Total and S1P H4 levels in nuclear fraction of cells were detected by immunoblotting.

Histones H2A and H4 are cotranslationally processed by MetAP and NatD (Fig. 1A). We hypothesized that the recruitment of NatD to ribosomes may be facilitated via NAC, analogously to its role in the recruitment of NatA (*33*). To identify possible modes of interaction, we first screened the protein interaction database BioGRID (*37*) and found the NACβ subunit of heterodimeric NAC among the reported binders of NatD in human cells (*38*, *39*). Among other reported interactors of NatD, we also found the upstream enzyme MetAP1 (*40*, *41*), which can remove iMet from nascent H2A and H4 before Nt-acetylation. These data on protein-protein interactions suggest that NAC could recruit both NatD and MetAP1 to ribosomes, similar to how it organizes the formation of a multienzyme MetAP1-NatA complex on ribosome-nascent chain complexes (RNCs) (*33*).

To test this hypothesis, we assembled the proposed RNC-NAC-MetAP1-NatD complex in vitro using purified components and imaged it by cryo–electron microscopy (cryo-EM). For complex reconstitution, we generated a translationally stalled human RNC bearing residues 2 to 37 of H4 (without iMet) followed by the modified Xbp1 arrest peptide (Fig. 1B). The 60–amino acid–long nascent chain sequence was designed such that the stalled RNC displays the N-terminal SGRG motif of H4—the substrate of NatD—outside the polypeptide tunnel exit, resembling the nascent histone chain cotranslationally processed by MetAP. We assembled the purified RNC_H4-60aa-SGRG_ with NAC, the catalytically impaired MetAP1 D220N mutant and wild-type NatD in the presence of CoA instead of Ac-CoA, aiming to capture the N terminus of the nascent polypeptide chain engaged by NatD but preventing its acetylation (Fig. 1A).

When processing the cryo-EM data, we identified stalled ribosomes with additional segments of density at the polypeptide tunnel exit through several rounds of three-dimensional (3D) classification (fig. S1). The resulting cryo-EM map was refined to an overall resolution of 3.55 Å with the local resolution of exit tunnel factors ranging from 4 to 8 Å (Fig. 1C, fig. S1 and S2, and table S1). In the cryo-EM map, the peptidyl-tRNA in the P-site of the 80S ribosome is linked to the Xbp1 arrest peptide in the exit tunnel, confirming the homogeneity of RNC stalling. The resolution of the additional density at the ribosomal tunnel exit was sufficient to confidently dock known structures of NAC, MetAP1, and NatD (Fig. 1, D and E, and fig. S2).

In the structure, the dimeric globular domain of NAC is bound to the 28*S* rRNA via its two positively charged α helices, placing it close to the ribosome tunnel exit, and the N-terminal basic motif of NACβ is wedged between eL19 and eL22, anchoring the heterodimer on the ribosome (Fig. 1, D and E). The globular C-terminal domain of MetAP1 occupies the position next to the globular domain of NAC (Fig. 1, D and E). These binding sites and orientations of NAC and MetAP1 on the ribosome are consistent with previous observations (*33*–*36*).

**Fig. 2.**
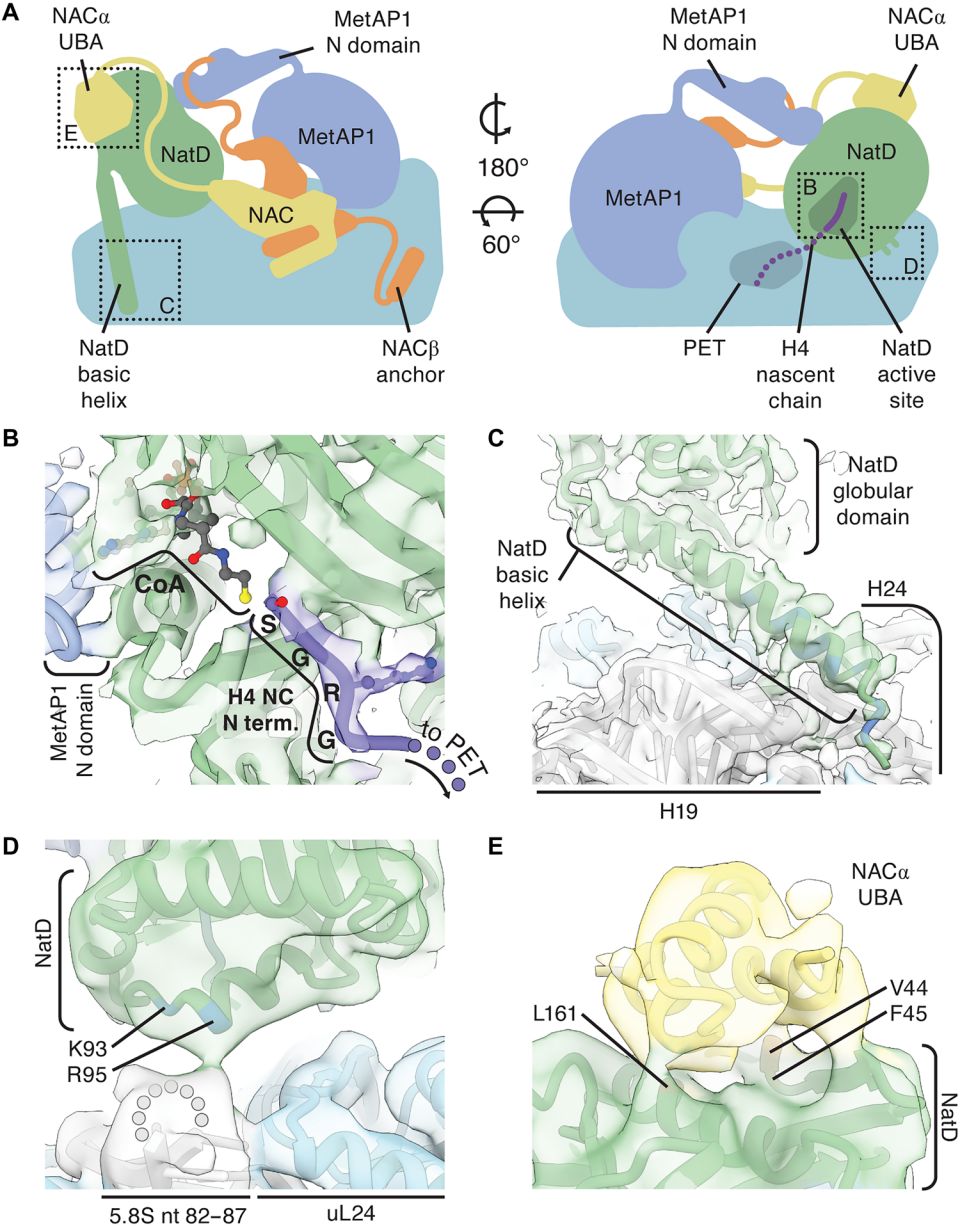
NatD binds RNC by contacting rRNA, nascent chain, and NAC. (**A**) Cartoon of NAC, MetAP1, and NatD bound at the ribosomal tunnel exit. Dotted boxes refer to structure details shown in (B) to (E). (**B**) The substrate-binding cavity of NatD accommodates the N-terminal SGRG motif of the nascent polypeptide and CoA. The CoA moiety beyond the phosphate adenosine diphosphate is not resolved in our map and is modeled as in PDB 4U9W. (**C**) The N-terminal basic helix of NatD binds to H19 and H24 of the 28*S* rRNA. (**D**) Minor basic patch of NatD is oriented toward the 82 to 87 nt loop of the 5.8*S* rRNA. (**E**) The UBA domain of NACα binds the globular domain of NatD. The main cryo-EM map [(B) and (D)] and the map low-pass filtered to 6 Å resolution [(C) and (E)] are superimposed on the model. The structure is colored as in Fig. 1. Positively charged amino acid residues are colored blue.

The density corresponding to ribosome-bound NatD extended beyond the known crystal structure of the truncated form of the protein (*14*), allowing us to model its previously unresolved N and C termini using the AlphaFold prediction as a starting model (fig. S3). In the substrate-binding pocket of RNC-bound NatD, we found a well-defined segment of cryo-EM density corresponding to the N-terminal SGRG motif of the H4 nascent chain (Figs. 1E and 2, A and B). The conformation of the captured nascent chain terminus matches that of the substrate in the crystal structure of NatD-CoA-H4 (*14*). Toward the other end of the substrate cavity, the bound CoA molecule is only partially visible in the cryo-EM map. While there is density for the 3′-phosphate adenosine diphosphate moiety (Fig. 2, A and B), the remaining part of the cofactor was not visible and was modeled as in the known structure of NatD-CoA-H4 (*14*).

We identified two additional segments of density adjacent to the globular domain of NatD, which could not be attributed to the unmodeled segments of the acetyltransferase. A small three-helix bundle and an extended fragment of density bridging the globular domains of MetAP1 and NatD were visible in the low-pass–filtered map of the complex (fig. S2). Guided by AlphaFold predictions and previously published data, we identified and modeled these parts of the complex. The three-helix bundle in the EM map matches the position and conformation of the NACα UBA domain in the AlphaFold prediction of the NatD-NAC complex (fig. S3). Binding of UBA to NatD suggests its role in the enzyme recruitment, similar to how it recruits NatA (*33*). The density bridging MetAP1 and NatD accommodates well the AlphaFold model of the N-terminal zinc finger domain of MetAP1 with bound C terminus of NACβ (*35*), which was incorporated into our model (Fig. 1, D and E, and fig. S2). This domain was previously not resolved in mammalian RNC-NAC-MetAP1 and RNC-NAC-MetAP1-NatA structures, possibly because it is conformationally dynamic (*33*–*35*). Although the N domain of MetAP1 is visible only at lower resolutions, we opted to model it in complex with the fragment of the C-terminal tail of NACβ, based on the previously reported functional and biochemical evidence indicating that the contact with NACβ is required for MetAP1 recruitment (*35*).

MetAP1 and NatD, bound on opposite sides of the globular domain of NAC, are positioned close to the polypeptide exit tunnel (PET) with both enzymes’ active sites oriented toward where nascent proteins emerge (Fig. 1D). The MetAP1 and NatD active sites are at equal distances from the PET opening—a much more compact architecture with less space available to the nascent polypeptide outside PET compared to the quaternary RNC-NAC-MetAP1-NatA complex, in which the Nt-acetyltransferase processes a much broader range of substrates. As the N domain of MetAP1 docks on NatD, it further confines the reaction space and possibly creates a functional link between the enzymes.

To test whether NAC helps recruit NatD to ribosomes, we developed a binding assay based on Förster resonance energy transfer (FRET) between a donor dye labeled at residue 12 in the H4 nascent chain on the RNC and an acceptor dye labeled on a ybbR tag (*42*, *43*) inserted at residue 223 near the substrate cavity of NatD (fig. S4). Measurements were carried out using RNC bearing residues 2 to 71 of the H4 nascent chain to mimic a cotranslational substrate for NatD after iMet excision. A FRET efficiency of 40% was detected between the dye pair with NAC-bound RNC_H4-70aa-SGRG_ (fig. S5). Equilibrium titrations using this FRET assay showed that NatD associates with H4 RNC with modest affinity, with an equilibrium dissociation constant (*K*_d_) of 143 nM. The presence of NAC enhanced NatD binding affinity nearly 20-fold, lowering the *K*_d_ to 8.7 nM (Fig. 1, F and G). Competition of this FRET signal with unlabeled wild-type NatD yielded a *K*_i_ value of 4.3 nM for the binding of wild-type NatD with RNC_H4_ (table S2), indicating that the ybbR insertion and fluorescence labeling did not substantially perturb the substrate binding affinity of NatD. These results support a role of NAC in assisting the recruitment of NatD to H4-translating ribosomes.

In agreement with the biochemical measurements, knockdown of NACα strongly reduced the ribosome association of NatD in human cells (Fig. 1H). Moreover, levels of histone H4 phosphorylation at serine 1, a histone modification down-regulated by the N-terminal acetyl group and mediated by Casein kinase 2α (CK2α) (*23*, *25*), increased strongly after knockdown of NACα (Fig. 1I, right), similar to knockdown of NatD (Fig. 1I, left). Together, these results suggest that NAC facilitates NatD recruitment and activity on the ribosome in vivo.

### NatD is recruited via interactions with rRNA and the NACα UBA domain

NatD interacts with the ribosome by contacting rRNA with its two positively charged patches (Fig. 2A). The first patch comprises a long N-terminal α helix of NatD rich in positively charged amino acid residues—a part of α0 of the NatD-specific N-terminal extension (*14*). This helix lodges over H19 and H24 of 28*S* rRNA (Fig. 2C), the same site where the N-terminal basic patch of NatA auxiliary subunit Naa15 binds (fig. S6). The second patch is a small basic region comprising residues K93 and R95 on the opposite side of the globular domain of NatD, which point toward the 82 to 87 nt loop of the 5.8*S* rRNA (Fig. 2D). These two contacts orient NatD on the ribosome to help position its substrate peptide-binding cavity over the polypeptide tunnel exit.

**Fig. 3.**
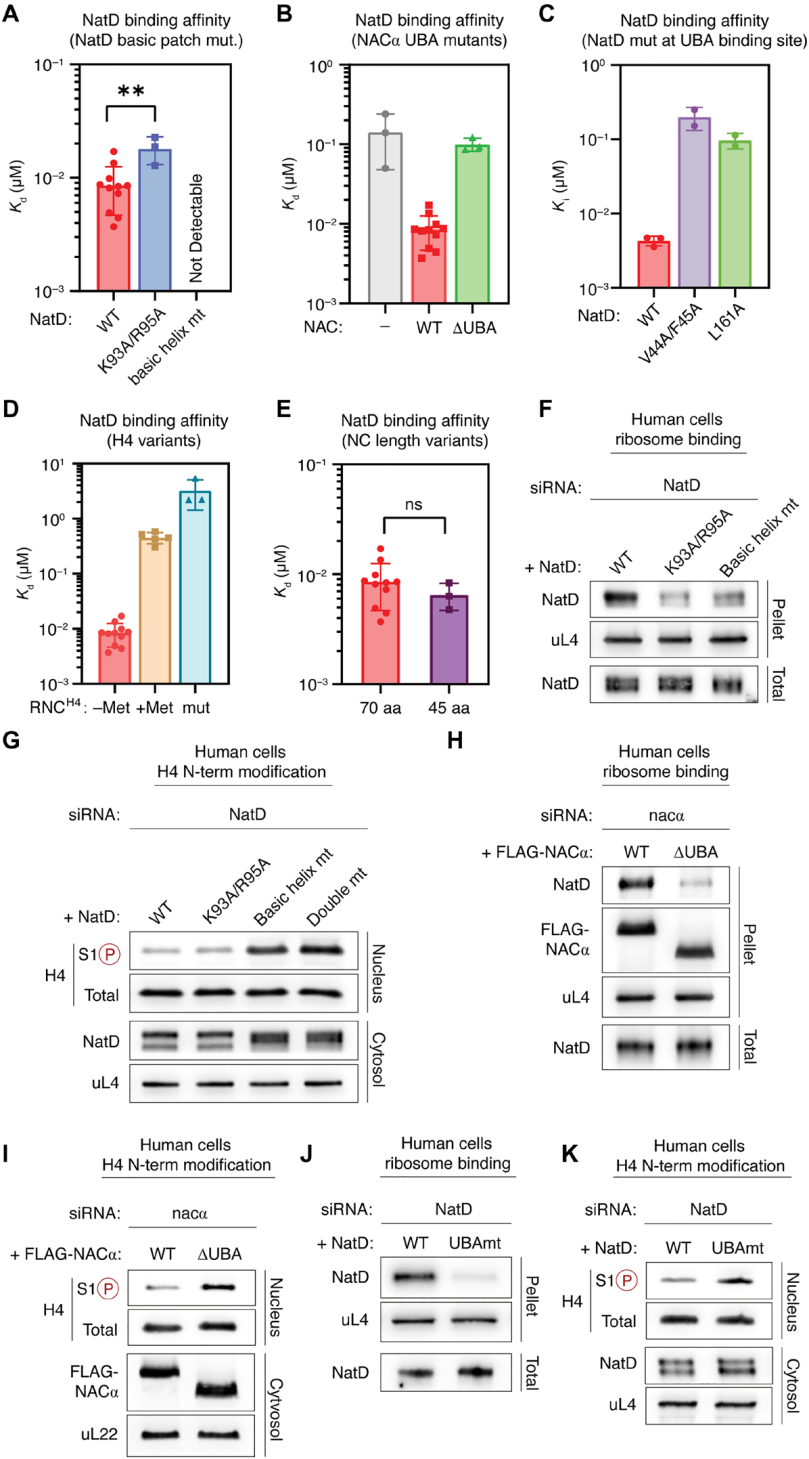
Disruption of contacts with the ribosome and NAC impairs NatD recruitment. (**A** to **E**) Summaries of the binding affinities (*K*_d_ or *K*_i_ values) of NatD for NAC-bound RNC with the indicated mutations in the NatD basic patch (A), the NACα UBA domain (B), and the NatD interaction interface with NAC UBA (C), and with changes in the N-terminal sequence (D) or length (E) of the H4 nascent chain. Values are obtained from the data in fig. S5 and are shown as means ± SEM, with the dots showing the value from independent measurements. Unpaired *t* test is used for analyzing the statistical significance of differences in *K*_d_ or *K*_i_ values. ** indicates *P* ≤ 0.01, and ns indicates *P* > 0.05. The *K*_d_ or *K*_i_ values and the number of replicate measurements for each sample are also provided in table S2. (**F**) Ribosome association of indicated NatD variants in human HEK293T cells in the endogenous NatD siRNA knockdown background. (**G**) Analysis of the *N*-acetyl-antagonistic histone phosphorylation mark at serine 1 (S1P) of H4 in the presence of indicated NatD variants in human HEK293T cells. Total H4 and S1 phosphorylation levels in nuclear fraction of cells were detected by immunoblotting. (**H**) Ribosome association of NatD in the presence of indicated FLAG-tagged NACα variants in human HEK293T cells. (**I**) Analysis of the *N*-acetyl-antagonistic histone phosphorylation mark at serine 1 (S1P) of H4 in the presence of indicated FLAG-tagged NACα variants in human HEK293T cells. (**J**) Ribosome association of wild-type and UBAmt (V44A/F45A/L161A) NatD variants in human HEK293T cells in the endogenous NatD siRNA knockdown background. (**K**) Analysis of the *N*-acetyl-antagonistic histone phosphorylation mark at serine 1 (S1P) of H4 in the presence of indicated NatD variants in human HEK293T cells. Proteins in total, nuclear, and ribosomal fractions, as well as H4S1 phosphorylation levels, were detected by immunoblotting [(F) to (K)].

To functionally validate the role of these contacts, we tested how mutations of these basic regions affected NatD affinity for NAC-bound RNC_H4_ in the FRET-based binding assay. Mutation of the minor basic patch (K93A/R95A) weakened the binding of NatD to the RNC_H4-70aa-SGRG_ twofold and lowered the FRET efficiency ~40% after binding has saturated, indicating a role of this basic patch in positioning NatD with respect to the H4 N terminus on the ribosome (Fig. 3A, fig. S5, and table S2). The basic helix NatD mutant (^3^RKSSKAKEKKQKRLEER^19^/^3^AASSKAAEAAQAALEEA^19^) showed no detectable FRET with RNC_H4-70aa-SGRG_, which points to a considerably stronger disruption of binding (fig. S5). Extending these findings, the Nt-acetylation of histone H4—measured indirectly via the antagonistic S1 phosphorylation mark—was greatly impaired in cells expressing the basic helix mutant (Fig. 3G). The Nt-acetylation defect further increased in cells expressing a double NatD mutant with both basic patches mutated, indicating that the two ribosome-NatD contacts are important for NatD activity in cells. In agreement with this, ribosome association of both NatD mutants was notably reduced in cells compared to wild-type NatD (Fig. 3F). Together, these data highlight the importance of NatD-rRNA contacts for functional docking of the enzyme on the ribosome.

Our structural model reveals that the contact of NatD with NAC is formed by the same face of NACα UBA that interacts with SRP and NatA. To understand the role of this interface in NatD recruitment to ribosomes, we generated NACα and NatD mutants designed to disrupt the UBA-NatD interface based on the structural data (Fig. 2E). We generated a NAC variant lacking the UBA domain (ΔUBA) as well as NatD variants with mutations in its UBA-binding surface (V44A/F45A and L161A or V44A/F45A/L161A). We then assessed the effects of these mutations in biochemical assays and in cultured cells.

In the FRET-based in vitro binding assay, deletion of the UBA domain of NACα raised the *K*_d_ for NatD binding affinity ~10-fold, to values observed in the absence of NAC. In addition, this mutation lowered the FRET efficiency nearly 50% even after binding has saturated (Fig. 3B, fig. S5, and table S2), indicating suboptimal positioning of the H4 N terminus with respect to NatD. Mutation of the hydrophobic patch on the UBA-binding surface of NatD (V44A/F45A and L161A) weakened NatD’s RNC binding affinity by more than 20-fold (Fig. 3C and fig S5). These results indicate a critical role of the NAC UBA-NatD contacts in high-affinity binding and precise positioning of the enzyme on the RNC_H4_.

Consistent with the in vitro binding affinity measurements, ribosome binding and Nt-acetylation of histone H4 was markedly impaired in cells upon mutation of the NACα-UBA-NatD binding interface (NACα ΔUBA and NatD V44A/F45A/L161A) (Fig. 3, H to K). We therefore conclude that the flexibly tethered UBA domain of NAC binds NatD to recruit it to translating ribosomes and enable Nt-acetylation of nascent histones H2A and H4.

### Nascent chain sequence dictates NatD binding to RNCs

The active site of ribosome-bound NatD is positioned approximately 4 nm away from the opening of the polypeptide tunnel exit—at a similar distance to that of the active site of MetAP1 (4 to 5 nm) and closer than that to the active site of NatA (6 nm) in the RNC-NAC-MetAP1-NatA/E structure (fig. S7). This spatial arrangement suggests that the onset of cotranslational nascent chain processing by the functionally docked NatD could occur at nascent chain lengths similar to that for MetAP1 (~60 amino acids) and shorter than that for NatA (75 to 100 amino acids) (*33*). Given the small size of histones, this compact architecture could ensure their timely Nt-acetylation by NatD before the termination of translation. In support of an early action of NatD, FRET-based binding assays showed that an RNC_H4_ bearing a 45–amino acid–long nascent chain bound NatD with the same affinity as that for the RNC_H4-70aa-SGRG_ (Fig. 3E, fig. S5, and table S2).

The well-resolved N-terminal SGRG motif of the H4 nascent chain in the substrate cavity of NatD in the structure indicates that the mode of NatD association with the ribosome is conducive to binding of the nascent chain N terminus that emerges from the ribosomal tunnel. To understand the role of the nascent chain sequence in NatD recruitment, we carried out FRET-based binding assays between NatD and NAC-bound RNCs bearing different sequence variations in the H4 nascent chain. We first tested the importance of the N-terminal SGRG motif by replacing it with ACAR, derived from the corresponding residues in the uL4 protein unrelated to H2A and H4. This mutation largely abolished the RNC binding of NatD (Fig. 3D, fig. S5, and table S2), supporting the highly sequence-specific recognition of H2A and H4-translating ribosomes by NatD based on the SGRG recognition motif (*13*, *14*, *24*).

The high affinity of NatD interaction with RNC_H4_ raises questions about how MetAP1, which binds RNCs with much lower specificity, is not excluded by NatD for accessing the H2A and H4 nascent chains. We therefore tested the importance of iMet excision in NatD recruitment using RNC bearing an iMet-containing H4 nascent chain (MSGRG). The inclusion of iMet also nearly abolished NatD binding to RNC_H4-70aa-MSGRG_ (Fig. 3D and fig. S5). This strongly suggests that NatD recruitment is precisely timed during the sequential processing of the H4 nascent chain, occurring after MetAPs have completed iMet excision.

These results suggest that efficient recruitment of NatD to histone RNCs is highly dependent on sequence-specific interactions with the N-terminal sequence and occurs in an ordered manner following iMet excision by the MetAPs. Furthermore, the architecture of the RNC_H4_-NAC-NatD complex enables NatD to engage shorter nascent chains on the ribosome compared to other NATs.

### MetAP1 and NatD cooperate during processing of nascent histones

**Fig. 4.**
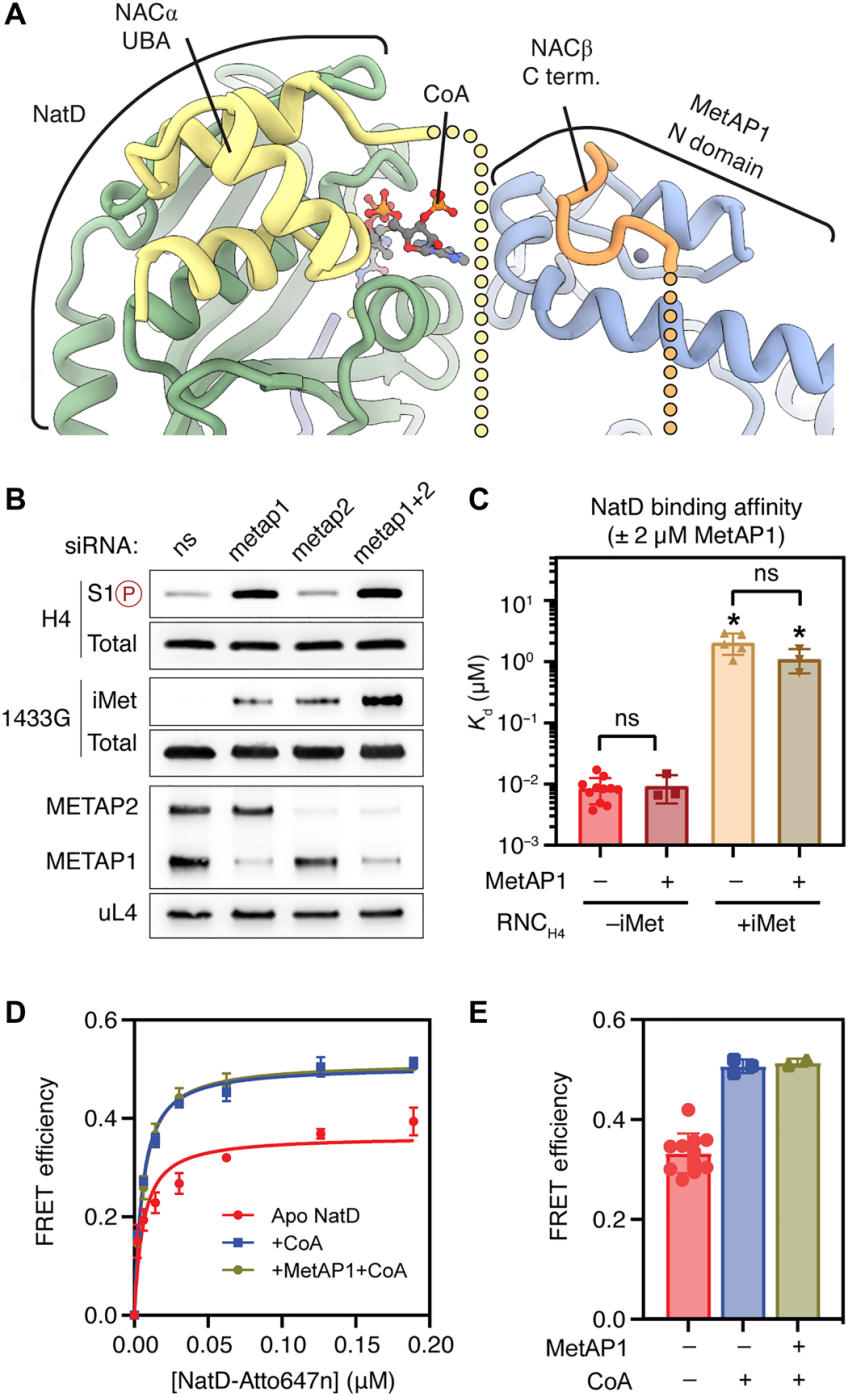
The N-terminal zinc finger domain of MetAP1 contacts NatD when both enzymes cobind on the ribosome. (**A**) The N domain of MetAP1 closes over the CoA-binding cavity of NatD. (**B**) N-terminal processing defects of H4 and 1433G after knockdown of METAP1 and METAP2 in human HEK293T cells. *N*-acetyl-antagonistic H4 phosphorylation mark at serine 1 (S1P, top panel) and 1433G still containing the initiator methionine (iMet, middle panel) were detected by immunoblotting using epitope-specific antibodies. Knockdown efficiencies of METAPs were analyzed by immunoblotting (bottom panel). (**C**) Summary of *K*_d_ values for NatD binding with NAC-bound RNC_H4_ in the presence and absence of 2 μM MetAP1, obtained from equilibrium titrations in fig. S5. Values are shown as means ± SEM, with the dots showing the values from independent titrations. * indicates that lower limits of *K*_d_ were reported. Unpaired *t* test is used for analyzing the statistical significance of differences in *K*_d_ values. ns indicates *P* > 0.05. (**D** and **E**) Equilibrium titrations to measure the binding affinity of NatD for NAC-bound RNC H4 in the presence and absence of CoA and MetAP1 (D). The lines are fits of the data to Eq. 2. The obtained *K*_d_ values are summarized in Table S2. The FRET efficiency in the RNC-NatD complex is summarized in (E). Values are shown as means ± SEM, with the dots showing the values from independent titrations. The *K*_d_ or *K*_i_ values and the number of replicate measurements for each sample are also provided in table S2.

The N-terminal zinc finger domain of MetAP1 binds NatD close to the CoA-binding cavity and caps the space over the PET between the globular domains of MetAP1 and NatD (Figs. 1, D and E, and 4A), creating an additional bound for the reaction space. The contact between MetAP1 and NatD could facilitate substrate handover between the enzymes and binding of NatD to substrate RNCs following iMet excision or have other functional roles in the sequential processing of nascent histones. Collectively, these observations led us to hypothesize that MetAP1 and NatD cooperate on the ribosome to process nascent histones.

To probe whether the NatD-MetAP1 contact could modulate the recruitment of NatD to histone RNCs, we performed a FRET-based binding assay with RNC_H4-70aa-SGRG_, NAC, and NatD in the presence of MetAP1. The additional presence of MetAP1 did not enhance the binding of NatD to RNC_H4-70aa-SGRG_, yielding a *K*_d_ of 11 nM similar to that without MetAP1 (Fig. 4C, fig. S5, and table S2). We further tested whether the N domain of MetAP1 could facilitate NatD prepositioning on histone RNCs before iMet excision. However, NatD binding to the iMet-containing H4 RNC_H4-70aa-MSGRG_ remained weak and was not enhanced by the additional presence of MetAP1 (Fig. 4C, fig. S5, and table S2).

Because the N domain of MetAP1 closes over the Ac-CoA-binding cavity of NatD (Fig. 4A), it could favor the recruitment of NatD charged with one of its substrates to histone nascent chains. We therefore tested whether addition of CoA altered the affinity of NatD for the RNC_H4_-NAC-MetAP1 complex. The presence of CoA did not affect the binding affinity of NatD but enhanced the FRET efficiency in the assembled complex, suggesting communication between the two substrate binding sites (Fig 4, D and E). However, the effect of CoA was observed with or without the presence of MetAP1. Therefore, while the structure indicates that the two enzymes form an architecturally connected space for the successive reactions to take place, they do not bind ribosomes cooperatively.

We therefore tested potential functional cooperation of NatD and MetAP1. We noted that the proposed mechanism of cooperation between these two enzymes is not expected for MetAP2, which lacks the N-terminal zinc finger domain and processes iMet independently of NAC (*44*). This model thus predicts that MetAP1 is privileged over MetAP2 to mediate the efficient sequential processing of the histone nascent chain.

To test this hypothesis, we assayed how the knockdown of MetAP1 or MetAP2 in human cells affects the N-terminal processing of histone H4 (Fig. 4B). Depletion of MetAP1—but not MetAP2—led to hyperphosphorylation of H4S1, suggesting retention of iMet on the NatD substrate. In contrast, methionine excision of a model NatA substrate (1433G-GFP) was equally impaired by knockdown of MetAP1 and MetAP2. These data suggest that NatD exclusively cooperates with MetAP1 to process the N termini of histones H4 and H2A, while NatA can cooperate with both MetAPs. In contrast to NatA, the cobinding of NatD and MetAP2 on the ribosome is sterically impossible because the binding sites of the two enzymes overlap (fig. S8). Furthermore, these data align well with the previous report that MetAP2 poorly processes MS-, MP-, and MA-starting proteins compared to MetAP1 (*45*). Combined, these observations suggest that MetAP1 is the main MetAP responsible for processing histones H2A and H4.

Together, these data help define the structural interface between MetAP1 and NatD, suggest that MetAP1 is the sole MetAP to produce NatD substrates by excising iMet from nascent histones H2A and H4, but show that the subsequent NatD recruitment is MetAP1 independent.

## DISCUSSION

In this study, we have uncovered the basis of cotranslational Nt-acetylation of histones H2A and H4 by NatD. Our findings demonstrate that ribosome-bound NAC recruits NatD via the flexible C-terminal tail of NACα. The UBA domain of NACα, electrostatic interactions with the ribosomal surface, and the conserved SGRG motif at the N terminus of H2A and H4 histones together drive the high-affinity binding of NatD to ribosomes and to functionally dock the enzyme at the PET to modify nascent histones. Moreover, our structure shows that NatD can engage substrate/histone RNCs alongside the upstream enzyme MetAP1, which contacts NatD with its N-terminal domain.

One of NatD’s basic patches interacting with the ribosome—the N-terminal basic helix—is a part of the larger N-terminal extension unique to single-subunit NatD (*9*, *14*). Its direct binding to rRNA sets NatD apart from heteromeric NATs, which bind ribosomes primarily via auxiliary subunits (*33*, *34*, *46*–*48*), and allows NatD to position the active site closer to the polypeptide tunnel exit. Alternative splicing or alternative translation initiation during NatD synthesis can produce a short isoform of NatD lacking the basic N-terminal fragment (*38*, *49*). We hypothesize that the short isoform has lower affinity for ribosomes and is likely to acetylate histones posttranslationally in the cytosol or in the nucleus.

The active site of ribosome-bound NatD is positioned approximately 4 nm away from the opening of the PET—at a similar distance to the active site of MetAP1 but closer than the active site of NatA in the RNC-NAC-MetAP1-NatA/E complex structure (*33*, *34*). This, together with NatD’s ability to bind nascent chains as short as 45 amino acids, should allow the enzymes to process histones in quick succession. Such architecture of the complex is likely dictated by the nature of NatD substrates: Composed of 130 and 103 amino acids, respectively, histones H2A and H4 are relatively short proteins and, thus, have a narrow window of opportunity to be cotranslationally processed before they are released from the ribosome.

Consistent with its substrate specificity (*13*, *14*), NatD recruitment to ribosomes is greatly influenced by the sequence of the translated protein. In vitro, NatD tightly binds only the RNCs with the substrate motif SGRG at nascent N termini, while RNCs with the nonsubstrate nascent chain and with iMet retained at the H4 N terminus strongly interfere with NatD binding. Our cell biology data indicate that MetAP1 is the only upstream enzyme generating NatD substrates, while the cryo-EM data reveal that MetAP1 and NatD can cobind on ribosomes and that the two enzymes interact with each other, whereas cobinding with MetAP2 is likely to be sterically impossible. Together, these observations suggest a cooperation between MetAP1 and NatD during nascent histone processing. In addition, the data on selective processing of H2A and H4 by MetAP1 suggest that the slow G_2_-M transition upon inhibition or knockdown of MetAP1 could, in part, be due to impaired histone modification (*50*).

We explored the hypothesis that NatD might be recruited to substrate RNCs before iMet excision via the N domain of MetAP1, which bridges the two enzymes in our cryo-EM structure; however, the addition of MetAP1 did not enhance the affinity of NatD for iMet-containing RNC and similarly did not change the affinity of NatD for the substrate RNC. Nevertheless, the observed structural features could still indicate an important functional role for the N domain of MetAP1 in histone processing. For example, as the N domain of MetAP1 closes on the globular domain of NatD and caps the space between the two enzymes over the ribosomal tunnel, it could limit the space available to the nascent polypeptide chain outside the PET and consequently accelerate the transfer of the substrate polypeptide between the enzymes.

Across the sets of NatD binding assays, we observed variation in maximum FRET efficiencies between fluorescently labeled H4-RNCs and NatD. These could reflect changes in conformational dynamics of the complexes, such as differences in the orientation of the enzyme relative to substrate nascent chains. Overall, disruption of the UBA-NatD interaction and NatD minor basic patch or shortening of the nascent chain produced lower FRET efficiencies compared to the RNC_H4-70aa_-NAC-NatD complex. Maximum FRET efficiency increased upon the addition of CoA without affecting the *K*_d_, possibly due to closer positioning of NatD to the nascent chain N terminus. This suggests possible cooperation between the two substrates of NatD.

Despite having distinct enzyme architectures and substrate specificities, the single-subunit NatD and the heterodimeric NatA share the mode of interaction with the ribosome: Both enzymes attach with a positively charged patch to the same segment of the 28*S* rRNA—H19 and H24 (fig. S6)—and both are recruited via the UBA domain of NACα (*33*). These overlapping ribosome footprints and shared recruitment mechanisms likely render NatD and NatA binding mutually exclusive, thereby preventing the enzymes from competing for substrates while still permitting sequential action with MetAP1. At the same time, the role of NAC in NatD recruitment reinforces the model of the NAC code that defines the processing path of newly synthesized proteins (*30*). For ER-targeted proteins, translation of the signal sequence leads to detachment of the globular domain of NAC from the ribosome to disfavor enzymatic modification of N termini and allow SRP binding (*36*). In contrast, translation of cytosolic and nuclear proteins permits the docking of NAC globular domain at the ribosomal tunnel exit, which supports N-terminal modification by a cohort of enzymes: MetAP1 (*35*), NatA (*33*), NMT1 (*51*), NMT2 (*52*), and—as shown in this study—NatD.

**Fig. 5.**
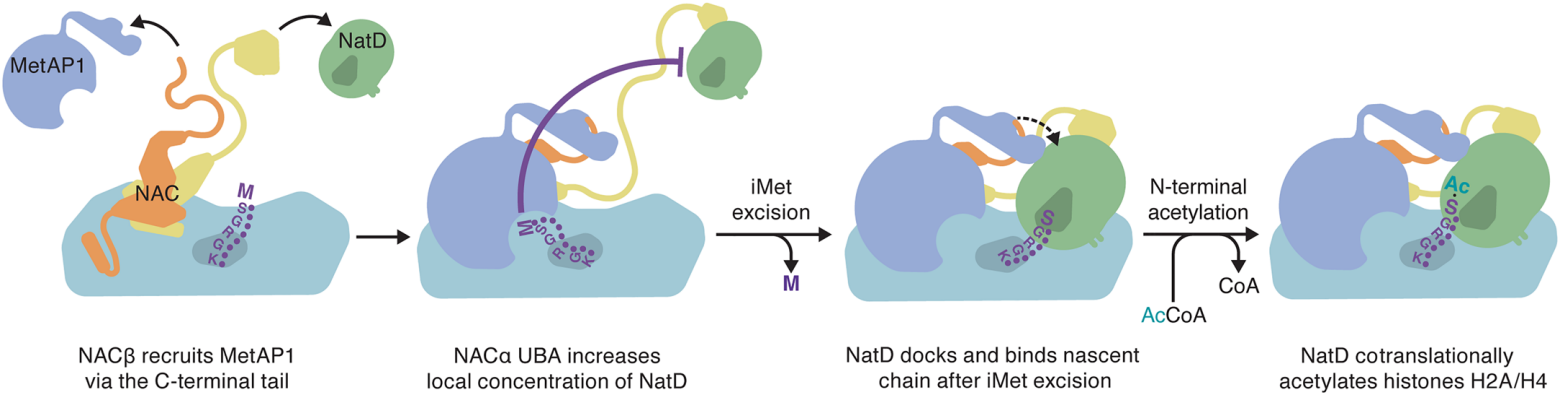
Model for recruitment of MetAP1 and NatD and cotranslational processing of histones H2A and H4.

Together, these data led us to put forth a model for sequential cotranslational processing of nascent histones H2A and H4 (Fig. 5). First, NAC recruits MetAP1 with the C terminus of NACβ early during translation, and the enzyme docks on the ribosome close to the polypeptide tunnel exit. The UBA domain of NACα concurrently increases the local concentration of NatD, while the unprocessed nascent chain prevents it from tightly binding the ribosome. As MetAP1 excises iMet from the nascent histone chain and generates the substrate for the acetyltransferase, NatD can dock on the ribosome alongside MetAP1 to acetylate the N terminus of H2A/H4. The magnitude of affinity increase of NatD for substrate versus nonsubstrate RNCs likely minimizes nonproductive interactions with ribosomes during the synthesis of ~200,000,000 histones H2A and H4, most of which are N-terminally acetylated over the course of the S phase. It is plausible that cotranslational histone maturation involves yet unidentified factors that may additionally link MetAP1 and NatD recruitment to translation of histone mRNAs or perform roles beyond enzymatic modification of histone N termini, which were not included in our in vitro reconstitution or binding experiments. Overall, this work expands our understanding of the mechanism of cotranslational modification of nascent histones and highlights the role of NAC as a broad regulator of protein biogenesis on the ribosome.

## MATERIALS AND METHODS

### Protein expression and purification

#### NatD

The coding sequence for human NatD without the iMet (UniProt ID Q86UY6-1, residues 2 to 237) with an N-terminal SUMO tag was synthesized and inserted into pET28a expression vector in line with the N-terminal His_6_ tag by TWIST bioscience. The expression plasmid was electroporated into *Escherichia coli* LOBSTR (*53*), and transformants were inoculated into LB supplemented with kanamycin (50 μg/ml) and grown overnight at 37°C with shaking. The starter culture was diluted 1:250 in superbroth (SB) medium supplemented with kanamycin (50 μg/ml) and chloramphenicol (12.5 μg/ml), and cells were grown at 37°C with shaking to an OD_600_ (optical density at 600 nm) of 1.3, at which time culturing temperature was shifted to 18°C and protein expression was induced by the addition of isopropyl β-D-1-thiogalactopyranoside (IPTG) to 0.2 mM for 16 hours. All subsequent procedures were done at 4°C unless noted otherwise. Cells were harvested by centrifugation at 7000*g* for 15 min, resuspended in buffer 2A [25 mM tris, 800 mM KCl, 20 mM imidazole, 5% (v/v) glycerol, and 0.5 mM tris(2-carboxyethyl)phosphine (TCEP), pH adjusted with HCl to 8.3 at room temperature] supplemented with EDTA-free cOmplete protease inhibitor cocktail (Roche) and lysed by sonication. Cell lysate was cleared by centrifugation in an SS-34 rotor (Sorvall) at 20,000 rpm for 45 min. Protein was purified using a fast protein liquid chromatography (FPLC) system AKTA pure (GE). Clarified lysate was applied to a 5-ml HisTrap FF column (GE); the column was washed with 10 column volumes (CV) of buffer 2A and 5 CV of buffer 2B [25 mM tris, 50 mM KCl, 20 mM imidazole, 5% (v/v) glycerol, and 0.5 mM TCEP, pH adjusted with HCl to 8.3 at room temperature]. Bound proteins were eluted directly onto a 5-ml HiTrap Q column (GE) with 5 CV of buffer 2C [25 mM tris-HCl, 50 mM KCl, 250 mM imidazole, 5% (w/v) glycerol, and 0.5 mM TCEP, pH adjusted with HCl to 8.3 at room temperature], and the column was washed with 5 CV of buffer 2B. His_6_-SUMO-NatD was eluted from the Q column with a linear gradient to 100% buffer 2A over 6 CV. Eluate fractions containing His_6_-SUMO-NatD were pooled and His_6_-SUMO tag was cleaved with SUMO protease ulp1 (produced in-house) for 30 min at 30°C. Protein precipitate formed during cleavage was removed by passing the solution through a 0.2-μm pore filter. NatD was separated from cleaved tag and His_6_-tagged ulp1 by reverse IMAC with a 5-ml HisTrap FF column. Flow-through fractions containing NatD were pooled, and the protein was transferred into the NatD storage buffer [25 mM tris, 200 mM KCl, 5% (v/v) glycerol, and 0.5 mM TCEP, pH adjusted with HCl to 8.3 at room temperature] using a 10,000 molecular weight cutoff centrifugal filter (Amicon). NatD aliquots were flash frozen in liquid nitrogen and stored at −80°C. Protein concentration was determined using extinction coefficient ε_280_ = 37,930 M^−1^ cm^−1^.

#### NAC and MetAP1

NAC and MetAP1 were expressed and purified as described previously (*33*).

#### Anti-GFP nanobody, BirA biotin ligase, and SENP^EuB^ protease

Anti-GFP (green fluorescent protein) nanobody for tag-off purifications, biotin ligase, and SENP^EuB^ protease were purified as described in (*54*) with slight modifications. Briefly, expression plasmids for His_14_-Avi-45xGS-anti-GFP nanobody (pTS117), His_14_-bdNEDD8-BirA (pTP264), and His_14_-TEV-SENP^EuB^ protease (pAV0286) were electroporated into *E. coli* LOBSTR (*53*), and transformants were inoculated into LB supplemented with kanamycin (50 μg/ml) and grown overnight at 37°C with shaking. Starter cultures were diluted 1:250 in SB medium supplemented with kanamycin (50 μg/ml) and chloramphenicol (12.5 μg/ml), and cells were grown at 37°C with shaking to an OD_600_ of 2.0, at which time culturing temperature was shifted to 18°C and protein expression was induced by the addition of IPTG to 0.2 mM for 16 hours. All subsequent procedures were done at 4°C unless noted otherwise. Cells were harvested by centrifugation at 7000*g* for 15 min, resuspended in lysis buffer (50 mM tris, 300 mM NaCl, 20 mM imidazole, and 0.5 mM TCEP, pH adjusted to 7.5 at room temperature using HCl), and lysed by sonication. Cell lysates were cleared by centrifugation in an SS-34 rotor (Sorvall) at 20,000 rpm for 45 min. Proteins were purified using an FPLC system AKTA pure (GE). For each protein, cleared lysate was applied to a 5-ml HisTrap FF column (GE), then the column was washed with 20 CV of lysis buffer, 20 CV of high-salt buffer (50 mM tris, 1000 mM NaCl, 20 mM imidazole, and 0.5 mM TCEP, pH adjusted to 7.5 at room temperature using HCl), and 5 CV of lysis buffer. Bound proteins were eluted with imidazole elution buffer [50 mM tris, 300 mM NaCl, 500 mM imidazole, 10% (w/v) glycerol, and 0.5 mM TCEP, pH adjusted to 7.5 at room temperature using HCl], and protein-containing eluate fractions were pooled. His_14_-Avi-45xGS-anti GFP nanobody and His_14_-bdNEDD8-BirA were transferred into BirA/NB storage buffer (50 mM tris, 200 mM NaCl, 0.5 mM TCEP, and 250 mM sucrose, pH adjusted to 7.5 at room temperature using HCl) using a 5-ml HiTrap Desalting column (GE). Aliquots of purified proteins were flash frozen and stored at −80°C. Protein concentrations were determined using extinction coefficients ε_280_ = 1.528 (mg/ml)^−1^ cm^−1^ for His_14_-Avi-45xGS-anti-GFP nanobody; ε_280_ = 50,420 M^−1^ cm^−1^ for His_14_-bdNEDD8-BirA; and ε_280_ = 45,380 M^−1^ cm^−1^ for His_14_-TEV-SENP^EuB^ protease.

### RNC purification for cryo-EM

#### Biotinylation of anti-GFP nanobody

Anti-GFP nanobodies were biotinylated as described in (*54*). Briefly, biotinylation was done for 3 hours at 25°C with 63 μM purified anti-GFP nanobody and 1 μM BirA in biotinylation buffer (50 mM Hepes-KOH, pH 7.5, 100 mM NaCl, 10 mM ATP, 12.5 mM MgCl_2_, and 10 mM biotin). Biotinylated nanobody was transferred into BirA/NB storage buffer using a 5-ml HiTrap Desalting column (GE) to remove excess biotin. Aliquots of biotinylated nanobodies were flash frozen in liquid nitrogen and stored at −80°C.

#### Preparation of a human cell extract–based in vitro translation system

A human cell extract–based in vitro translation system was prepared as described in (*55*) with modifications. One volume of the HeLa cytoplasmic extract (IPRACELL) was thawed on ice and mixed with 0.013 volume of 11.65 mg/ml preparation of GADD34, incubated at 33°C for 5 min, then mixed with 0.11 volume of in vitro translation supplement (190 mM Hepes-KOH, pH 7.0, 1.5 M KCl, 11 mM MgCl_2_, and 250 mM creatine phosphate), flash frozen in liquid nitrogen, and stored at −80°C. The concentration of KCl in the supplement was increased compared to the published protocol to enhance translation of mRNAs with EMCV IRES.

#### In vitro transcription

The sequence for T7 RNA polymerase promoter followed by EMCV IRES and an open reading frame encoding a fusion protein composed of (from N to C terminus) EGFP, 22xGS linker, mutant SUMO tag (*54*), residues 2 of 37 of histone H4, modified Xbp1 arrest peptide (DPVPYQPPFLCQWGRHQCAWKPLM), VV linker, and a His_6_ tag was synthesized and inserted into pTwist CMV vector by TWIST bioscience. Template DNA for in vitro transcription of the mRNA was generated by PCR from the plasmid using Q5 DNA polymerase (NEB) to produce an amplicon containing a T7 polymerase promoter at the 5′ end, sequence for EMCV IRES, open reading frame, and an A_30_ tail after the stop codon introduced with the overhang on the reverse primer. The amplicon was purified from agarose gel using a Monarch DNA Gel extraction kit (NEB). Transcription reaction with DNA template (0.8 ng/μl), 5.8 μM T7 RNA polymerase (produced in-house), 5 mM of each NTP, 40 mM tris-HCl, pH 7.6, 45 mM MgCl_2_, 1 mM spermidine, 5 mM DTT, 0.1% (v/v) Triton X-100, and RNaseOUT ribonuclease inhibitor (0.08 U/μl; Invitrogen) was incubated for 3 hours at 37°C, then supplemented with RNase-free DNaseI (Qiagen) and incubated for 1 hour 37°C to digest the DNA template. Transcribed mRNA was precipitated with LiCl, resuspended in mQ water and stored at −20°C.

#### In vitro translation and RNC purification

Before the translation reaction, the mRNA was incubated for 5 min at 65°C then cooled on ice. Treated human cell lysate was thawed on ice, then 1 volume of the lysate was mixed with 0.2 volume of mRNA, with the final mRNA concentration of 125 nM. Reaction was incubated in a water bath at 33°C for 15 min, then transferred on ice. All subsequent procedures were performed at 4°C. For affinity purification of the stalled RNC bearing a GFP-SUMO tag from 1 volume of an in vitro translation reaction, we used 0.15 volume of streptavidin magnetic beads slurry (Pierce). First, magnetic beads were charged with biotinylated anti-GFP nanobodies (0.3 μg of nanobodies per 1 μl of magnetic beads slurry) and blocked with biotin [see (*54*) for detailed protocol]. Charged magnetic beads were resuspended in 1 volume of the translation reaction mixed with 1.5 volumes of buffer T1 [50 mM Hepes-KOH, pH 7.5, 100 mM KCl, 5 mM MgCl_2_, and 0.1% (v/v) Triton X-100] and rotated head-over-tail for 1 hour at 4°C. The beads were then washed with 2 reaction volumes of buffer T1, 2 reaction volumes of buffer T2 [50 mM Hepes-KOH, pH 7.5, 500 mM KCl, 5 mM MgCl_2_, and 0.1% (v/v) Triton X-100] and with 1 reaction volume of buffer T1. To elute bound RNCs, magnetic beads were resuspended in buffer T1 with 250 nM SENP^EuB^ protease and incubated for 20 min with occasional mixing. The eluate was centrifuged at 16,100*g* for 1 min to remove beads not captured on the magnet, layered over a 30% sucrose cushion prepared with buffer T1 and spun at 85,000 rpm in a TLA-100 rotor (Beckman Coulter) for 1.5 hours to pellet RNCs. Pelleted RNCs were resuspended in buffer RB [50 mM Hepes-KOH, pH 7.5, 150 mM KOAc, pH 7.45, 5 mM Mg(OAc)_2_, and 0.5 mM TCEP], flash frozen in liquid nitrogen, and stored at −80°C.

### Cryo-EM sample preparation

#### Reconstitution of macromolecular complexes for cryo-EM experiments

To reconstitute the quaternary RNC-NAC-MetAP1-NatD complex for cryo-EM samples, 100 nM RNC_H4-60aa_, 500 nM NAC, 1 μM MetAP1, and 1 μM NatD were combined in buffer RB supplemented with 0.02% octaethylene glycol monododecyl ether, 3 μM CoA, and 4 mM NaCl. NAC, NatD, and MetAP1 were added sequentially to the mixture with 10-min incubation at room temperature after the addition of every factor, then the sample was placed on ice until grid preparation.

#### Cryo-EM grid preparation and data collection

For cryo-EM sample preparation, Quantifoil R2/2 Cu 300 grids were washed with ethyl acetate, coated with a 1-nm-thick continuous layer of amorphous carbon (produced in-house), and glow-discharged for 15 s at 15 mA using PELCO easiGlow glow discharge cleaning system (Ted Pella). Grids were mounted into Vitrobot MK IV (Thermo Fisher Scientific) with the chamber set to 4°C and 95% humidity. Sample (3.5 μl) was applied to a grid, and the sample was incubated for 30 s in the Vitrobot chamber; excess sample was blotted off grids for 2 to 4 s with a blot force of 15, and grids were plunge frozen in a 1:2 mixture of ethane and propane (*33*).

Grids were imaged in a Titan Krios G4 transmission electron microscope operating at 300 kV and equipped with a BioContinuum (Krios G4) imaging filter-mounted K3 direct electron detector, operating in 2× binned super-resolution mode. The microscope was used with a nominal magnification of 81,000×. The energy filter slit was set to 20 eV, and the defocus was set to shift between −0.6 and −2.4 μm in 0.3-μm steps. Automated data collection in the aberration-free image shift mode was set up in EPU 3.6.0.6389 (Thermo Fisher Scientific). A total of 9263 movies were collected at a physical pixel size of 1.065 Å per pixel with the total electron dose of 50 e^−^ Å^−2^.

### Cryo-EM data processing

Cryo-EM movie frames were motion corrected, dose weighted, and summed into micrographs in CryoSPARC Live (*56*). Particles were picked in CryoSPARC Live using a circular blob (250 to 350 Å blob diameter range) as a reference. All subsequent processing steps were done in CryoSPARC. Picked particles were extracted with a box size of 560 pixels, Fourier cropped to a box size of 186 pixels, and subjected to 2D classification (number of classes set to 200). A total of 467,339 particles from ribosome 2D classes were selected for further processing. After a homogeneous refinement, the selected particles were reextracted at full box size and homogeneously refined again. Refined particles were assigned into exposure groups based on image shift applied during data collection and refined with enabled optimization of per-group CTF parameters and per-particle defocus. The resulting particles were subjected to 3D classifications to select translating ribosomes with a P-site tRNA and the Xbp1 arrest peptide in the PET (150,179 particles, approximately 30% of all ribosomes), and then out of those, the RNCs with either NAC-NatD or NAC-MetAP1 bound at the tunnel exit (focus masks covering NAC-NatD or NAC-MetAP1, respectively; nondefault parameters of 3D classifications are presented in fig. S1). Sets of particles corresponding to H4 RNC-NAC-NatD and H4 RNC-NAC-MetAP1 were intersected, and the resulting stack of 47,409 particles (31% of RNC_H4-60aa_ particles, and 10% of all ribosomes) was subjected to further 3D classification with a focus mask on NAC-MetAP1 to separate particles with distinct MetAP1 conformations (fig. S1 and fig. S7) and particles with poor signal for the catalytic domain of MetAP1. Two 3D classes with well-defined density for the MetAP1 catalytic domain showed this domain in two conformations: rotated either toward or away from the globular domain of NAC—with the former showing better resolved elements of secondary structure. We used the respective set of 11,206 particles (7.3% of RNC_H4-60aa_ particles, and 2.4% of all ribosomes) for the final round of refinement, resulting in a map with an overall resolution of 3.55 Å. The final map was low-pass filtered and filtered to estimated local resolution using CryoSPARC.

### AlphaFold prediction of the NACαβ-NatD complex structure

Prediction of a structure of the human NACαβ-NatD complex was done with ColabFold 1.5.2 (*57*) implementing AlphaFold multimer v3 (*58*, *59*) with default parameters. Amino acid sequences of NACα (sequence id Q13765), NACβ isoform 2 (sequence id P20290-2), and NatD (sequence id Q86UY6) were retrieved from the UniProt database (*60*).

### Molecular model building, refinement, and visualization

Models of the ribosomal subunits [40S from Protein Data Bank (PDB) 8PPK (*61*) and 60S from PDB 9GMO (*62*)], the P-site tRNA [from PDB 7O7Y (*63*)], the Xbp1 arrest peptide [from PDB 6R5Q (*64*)], the NAC heterodimer [from PDB 7QWR (*36*)], the catalytic domain of MetAP1 [from PDB 2B3H (*65*)], and NatD [PDB 4U9W (*14*)] were docked into the final cryo-EM density map as rigid groups. The docked models were manually adjusted in Coot (*66*) to better fit the cryo-EM density. The Met-tRNA structure was modeled using the sequence of the human tRNA-Met-CAT-6-1 from the genomic tRNA database (*67*), and a matching mRNA triplet was built in the P-site of the small ribosomal subunit. In the model of the nascent polypeptide, the Xbp1 arrest peptide was extended at its N terminus toward the exit of the ribosomal tunnel with the sequence of the nascent chain construct. The UBA domain of NACα (residues 175 to 215) was transplanted from the AlphaFold model of the NACαβ-NatD complex. The N-terminal domain of MetAP1 (residues 5 to 70) with the bound C-terminal segment of NACβ (residues 140 to 149) was transplanted from the AlphaFold model of the NACβ-MetAP1 complex (*35*). The N-terminal segment of NatD (residues 2 to 15) was transplanted from the AlphaFold model of the NACαβ-NatD complex, and the internal loop (residues 201 to 208) and the C-terminal tail (220 to 237) were built de novo. The fragment of the nascent chain comprising the N-terminal pentapeptide of histone H4 and coenzyme A bound in the active site of NatD were modeled as in PDB 4U9W with minor adjustments. The full model of the RNC_H4-60aa_-NAC-MetAP1-NatD was assembled in PyMOL 3.1.3 (The PyMOL Molecular Graphics System, Version 3.0 Schrödinger, LLC) and was subjected to two macrocycles of real-space coordinate and ADP refinement in Phenix 1.21.2-5419 (*68*) with enabled secondary structure restraints. Following minor corrections, the model was again subjected to two macrocycles of real-space coordinate and ADP refinement in Phenix. The final model was validated using the MolProbity (*69*) tool implemented in Phenix and shows good geometry and model-to-map fit (fig. S2 and table S1). The structure was visualized using ChimeraX ver. 1.4 (*70*) to generate figure panels for the manuscript.

### Fluorescence labeling

#### NatD labeling

The ybbR tag (DSLEFIASKLA) (*42*, *43*) replaced residues ^221^DS^222^ in NatD. NatD-ybbR was purified following the same procedure as for WT NatD. CoA conjugates of Atto647n were generated by reacting maleimide-conjugated dye with a 1.75 molar excess of CoASH in 25% dimethyl sulfoxide (DMSO) and 75% Na⋅phosphate (pH 7.0) while stirring in the dark for 1 hour at room temperature. CoA-dye conjugate was purified by high-performance liquid chromatography (HPLC) using a C18 wide pore column (Supelco Analytical) over a 0 to 100% acetonitrile gradient. Peak fractions were lyophilized for ~36 hours, and purified CoA-dye conjugate was resuspended in DMSO. NatD-ybbR (20 μM) was labeled in SFP labeling buffer (50 mM Hepes-KOH, pH 7.5, 10 mM MgCl_2_, and 15% glycerol) with His_6_-tagged SFP enzyme (8 μM) and a fourfold molar excess of CoA-dye conjugate for 2 hours in the dark at room temperature. His_6_-SFP was removed using Talon resin (Clontech). The flowthrough containing labeled NatD was separated from free dye using a 40-ml Sephadex G25 column (Cytiva) in gel filtration buffer (50 mM Hepes-KOH, pH 7.5, 300 mM NaCl, 2 mM 2-mercaptoethanol (βME), 0.04% Nikkol, and 20% glycerol), concentrated using an Amicon centrifugal filter, flash frozen in aliquots in liquid nitrogen, and stored at −80°C.

#### RNC labeling

RNC bearing residues 2 to 71 of the H4 nascent chain was labeled with Cy3B at residue 12. An amber codon was incorporated at residue 12 in the H4 coding sequence. Templates for in vitro transcription were generated by PCR, resulting in amplicons that included, from the 5′ to 3′ end, a T7 promoter, the EMCV IRES, and the coding sequence for a FLAG tag, SUMO, and H4 residues 2 to 71. Amplicons were purified using a Qiagen PCR purification kit. In vitro transcription, translation, and purification of the RNC were performed in RRL as described previously (*33*, *71*). To generate fluorescent RNC, an amber suppressor system [1 μM *Mm*PylRS, *Mm*PyltRNA (100 mg/liter), and 100 μM axial-trans-cyclooct-2-en-L-Lysine (TCOK) (SiChem)] was included during in vitro translation to incorporate the unnatural amino acid TCOK at the amber codon position. The resulting RNC was incubated with 2 μM tetrazine-conjugated Cy3B at 25°C for 20 min before immunopurification using anti-FLAG resin (*33*).

### Fluorescence measurements

All fluorescence measurements were carried out on a Fluorolog 3-22 or FluoroQM-75-22 spectrofluorometer (HORIBA) at 25°C in RNC buffer [50 mM Hepes-KOH, pH 7.5, 150 mM KOAc, 5 mM Mg(OAc)_2_, 5 mM DTT, and 0.02% octaethylene glycol monododecyl ether (Sigma-Aldrich)] supplemented with bovine serum albumin (1 mg/ml). Proteins were ultracentrifuged for 30 minutes at 100,000 rpm in TLA100 (Beckman Coulter) before all assays.

#### Fluorescence emission spectra

Spectra were recorded using an excitation wavelength of 535 nm and emission wavelengths from 550 to 720 nm. FRET between Cy3B-labeled H4 RNC (RNC^Cy3B^) and Atto647n-labeled NatD (NatD^Atto647n^) was detected using 2 nM RNC^Cy3B^, 100 nM NatD^Atto647n^, and 50 nM NAC, with or without 1 μM unlabeled NatD where indicated.

#### Equilibrium titrations

To measure the binding affinity between H4 RNC and NatD, equilibrium titrations were performed using 2 nM RNC^Cy3B^ or preformed RNC^Cy3B^-NAC complex, with sequential additions of the indicated concentrations of NatD^Atto647n^. Fluorescence emission of RNC^Cy3B^ was recorded using an excitation wavelength of 535 nm and an emission wavelength of 577 nm. A control titration with unlabeled NatD was carried out in parallel. Raw fluorescence intensities were corrected for dilution and buffer background, and observed FRET efficiencies (*E*) were calculated using Eq. 1

$$E=1-\frac{F_{\text{DA}}}{F_{D}}$$ (1)

in which *F*_DA_ and *F*_D_ are the donor fluorescence intensities in the titrations with labeled and unlabeled NatD, respectively. The NatD concentration dependence of *E* was fit to Eq. 2

$$E=E_{\text{max}}\times \frac{K_{d}+{\left[A\right]}_{0}+\left[T\right]-\sqrt{{\left(K_{d}+{\left[A\right]}_{0}+\left[T\right]\right)}^{2}-4{\left[A\right]}_{0}\left[T\right]}}{2{\left[A\right]}_{0}}$$ (2)

in which [A]_0_ and [T] are the total concentrations of analyte (RNC) and titrant (NatD), respectively, *E*_max_ is the FRET efficiency at saturating titrant concentrations, and *K*_d_ is the equilibrium dissociation constant. *K*_d_ was constrained to be greater than 0.1 nM during data fitting using Prism v10.1.0 (*33*).

#### Competition experiments

Increasing concentrations of unlabeled WT or mutant NatD (I) was added to a solution containing 2 nM RNC^Cy3B^ and 50 nM NatD^Atto647n^ (L). Observed FRET efficiency (*E*) was plotted as a function of I concentration and fit to Eq. 3

$$E=E_{\text{Max}}\times \frac{K_{i}\left(1+\frac{\left[L\right]}{K_{d,L}}\right)}{\left[I\right]+K_{i}\left(1+\frac{\left[L\right]}{K_{d,L}}\right)}$$ (3)

in which *E*_Max_ is the FRET efficiency in the absence of inhibitor, *K*_d*,*L_ is the *K*_d_ of NatD^Atto647N^ (set to 10 nM), and *K*_i_ is the inhibition constant for unlabeled NatD. *K*_i_ is equal to the *K*_d_ of unlabeled NatD for the H4 RNC.

### Human cell culture experiments

#### Cell culture and RNAi

HEK293T cells (RRID:CVCL_0063) were cultured in Dulbecco’s modified Eagle’s medium supplemented with 10% fetal calf serum and Normocin (100 μg/ml) in a 5% CO_2_ atmosphere at 37°C. Cells were transfected with DNA and/or siRNA by electroporation in OptiMEM using a NEPA21 electroporator (Nepagene). To knock down NACα and NatD, cells were transiently transfected with siRNA duplexes (1 μg) targeting the 3′ untranslated regions (3′UTRs) of the genes (NACα: 5′-AGGAGUAACUGCAGCUUGG-dTdT-3′ and NatD: 5′-UCACAAUGCUCUCUCCUAA-dTdT-3′). Control cells were transfected with nonsense siRNA (5′-UUCUCCGAACGUGUCACGU-dTdT-3′). NACα and NatD expression was restored in knockdown cells by cotransfection of plasmids expressing N-terminally 3×FLAG-tagged NACα variants (20 μg) and untagged NatD variants (2 μg), respectively, from the CMV promoter and SV40 3′UTR. Knockdown of METAP1 and METAP2 was performed similarly using the siRNAs 5′-GGAAACAUUGCUCAACUCU-dTdT-3′ and 5′-GAGCCUCAGUGGAUGAAGU-dTdT-3′, respectively. All cell culture experiments were repeated at least three times as biological replicates.

#### Ribosome cosedimentation assays

Ribosome binding studies were performed as previously described (*33*, *35*). In brief, cells were harvested 2 days after transfection and extracted in ice-cold lysis buffer [30 mM Hepes, pH 7.4, 100 mM KOAc, 5 mM MgCl_2_, 5% Mannitol, 0.04% octaethylene glycol monododecyl ether, cycloheximide (100 μg/ml), 1 mM DTT, and 1× protease inhibitor cocktail (Roche)]. Ribosomes were pelleted by ultracentrifugation (220,000*g*) through a 25% sucrose cushion (prepared in lysis buffer) for 1.5 to 2 hours at 4°C. Proteins in the ribosomal pellet and total fractions were then analyzed by standard SDS–polyacrylamide gel electrophoresis (SDS-PAGE) and immunoblotting techniques.

#### Detection of H4S1 phosphorylation

To analyze defects of histone H4 N-acetylation in NACα and NatD mutant cells, the *N*-acetyl-antagonistic histone phosphorylation mark at serine 1 of H4 (*23*, *25*) was examined 2 days after transfection of siRNAs and rescue plasmids. Cells were lysed in NP40 lysis buffer [50 mM Na-PO_4_, pH 7.5, 150 mM NaCl, 2 mM EDTA, 1 mM EGTA, 0.5% NP40, and 1× protease inhibitor cocktail (Roche)] on ice for 5 min, and the cell nuclei were pelleted by centrifugation (20,000*g*, 5 min, 4°C). Supernatant (cytosolic fraction) was carefully removed, and the pellet (nuclear fraction) was extracted by boiling (99°C, 5 min) in SDS buffer [125 mm tris, pH 6.8, 2 mM EGTA, 4% SDS, and 1× protease inhibitor cocktail (Roche)]. The denatured nuclear fraction was then sonicated to shear the DNA and boiled again for 5 min at 99°C. Cytosolic and nuclear fractions were then analyzed using standard SDS-PAGE and immunoblotting techniques.

#### Detection of MetAP activity

To measure METAP activity in cells, cells were cotransfected with a plasmid (5 μg) expressing the first 12 N-terminal residues of human 1433G (^1^MVDREQLVQKAR^12^) fused to GFP under the eEF1A promoter [pEF-BOS vector (*72*)]. Reporter levels still containing the N-terminal methionine were then detected by immunoblotting using an epitope-specific monoclonal antibody (Novus Biologicals), as previously described (*35*). The immunoblots were then reprobed with an anti-GFP antibody (Roche) to detect the total amount of substrate (see antibodies details below).

### Antibodies

The following antibodies were used to detect proteins by immunoblotting: mouse anti-FLAG (Sigma-Aldrich, catalog no. F3165, RRID:AB_262044), rabbit anti-H4 phospho-S1 (abcam, catalog no. ab177309, RRID:AB_2797594), mouse anti-H4 (Santa Cruz Biotechnology, catalog no. sc-377520, RRID:AB_3674828), rabbit anti-NatD (abcam, catalog no. ab106408, RRID:AB_10866699), rabbit anti-NatD (Proteintech, catalog no. 16698-1-AP, RRID:AB_3085505), mouse anti-uL4 (Santa Cruz Biotechnology, catalog no. sc-100838, RRID:AB_2181910), mouse anti-uL22 (Santa Cruz Biotechnology, catalog no. sc-515904, RRID:AB_3107048), mouse anti-Actin (Santa Cruz Biotechnology, catalog no. sc-47778, RRID:AB_626632), mouse anti-iMet-1433G (Novus Biologicals, catalog no. NB-100-407, RRID:AB_10003122), mouse anti-GFP (Roche, catalog no. 11814460001, RRID:AB_390913), rabbit anti-NACα (Biorbyt, catalog no. orb411671), rabbit anti-METAP1 (Bethyl Laboratories, catalog no. A305-584A, RRID:AB_2891500), and rabbit anti-METAP2 (Proteintech, catalog no. 17040-1-AP, RRID:AB_2144162).
