# Supplementary material for: Mechanism of cotranslational modification of histones H2A and H4 by MetAP1 and NatD
Source: Sci Adv. 2025 Dec 19;11(51):eaeb1017. doi: 10.1126/sciadv.aeb1017 (PMC12716425; doi:10.1126/sciadv.aeb1017)
Supplement: Supplementary file 1 — Figs. S1 to S8 Tables S1 and S2 [file sciadv.aeb1017_sm.pdf]

Science Advances
AAAS

Supplementary Materials for

Mechanism of cotranslational modification of histones H2A and H4 by MetAP1 and NatD

Denis Yudin *et al.*

Corresponding author: Nenad Ban, ban@mol.biol.ethz.ch; Shu-ou Shan, sshan@caltech.edu; Martin Gamerdinger, martin.gamerdinger@uni-konstanz.de

Sci. Adv. **11**, eaeb1017 (2025)
DOI: 10.1126/sciadv.aeb1017

This PDF file includes:

Figs. S1 to S8
Tables S1 and S2

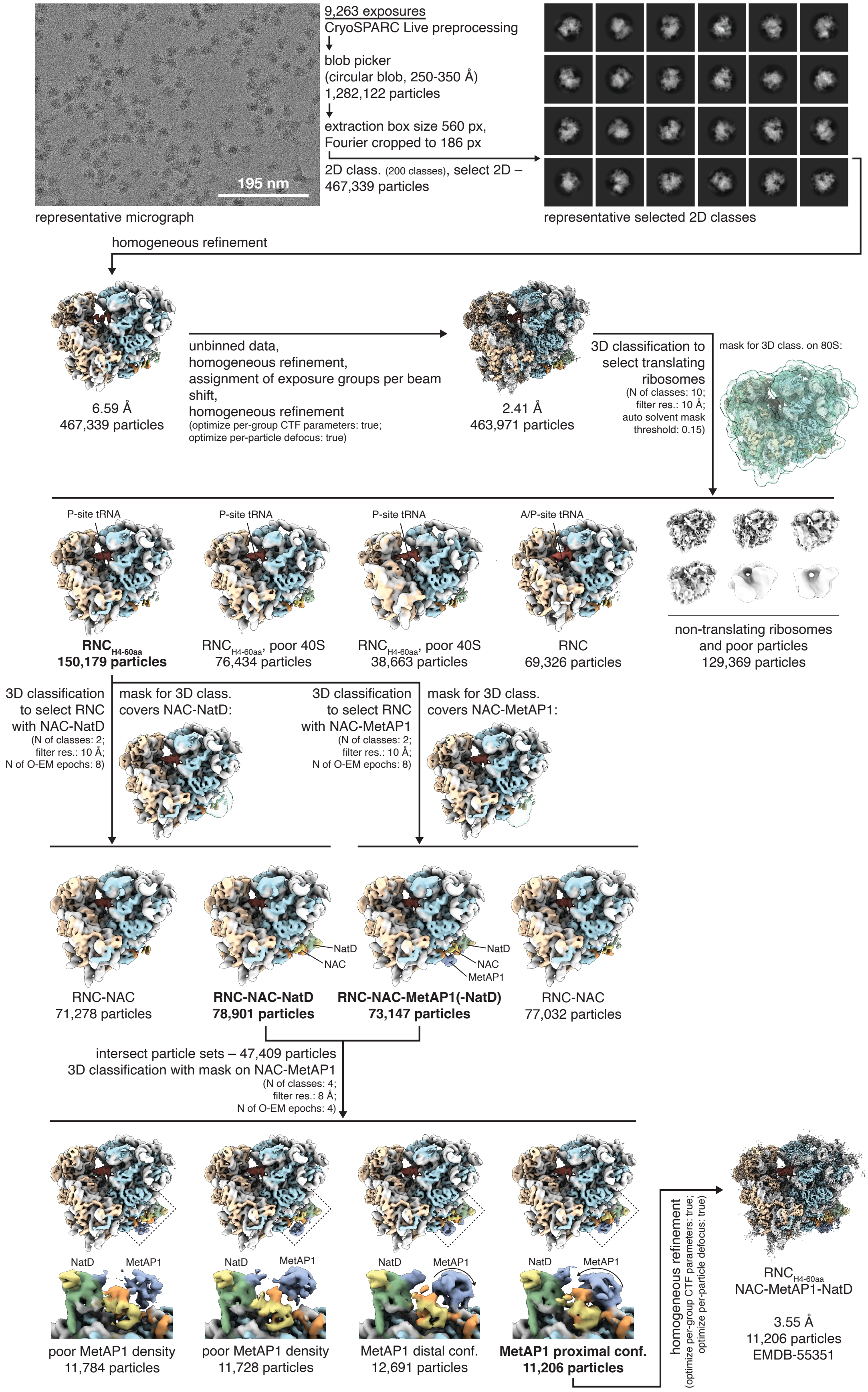
9,263 exposures
CryoSPARC Live preprocessing
blob picker
(circular blob, 250-350 Å)
1,282,122 particles
extraction box size 560 px,
Fourier cropped to 186 px
2D class. (200 classes), select 2D –
467,339 particles
195 nm
representative micrograph
representative selected 2D classes
homogeneous refinement
6.59 Å
467,339 particles
unbinned data,
homogeneous refinement,
assignment of exposure groups per beam shift,
homogeneous refinement
(optimize per-group CTF parameters: true;
optimize per-particle defocus: true)
2.41 Å
463,971 particles
3D classification to select translating ribosomes
(N of classes: 10;
filter res.: 10 Å;
auto solvent mask threshold: 0.15)
mask for 3D class. on 80S:
P-site tRNA
P-site tRNA
P-site tRNA
A/P-site tRNA
RNC$_{H4-60aa}$
150,179 particles
RNC$_{H4-60aa}$, poor 40S
76,434 particles
RNC$_{H4-60aa}$, poor 40S
38,663 particles
RNC
69,326 particles
non-translating ribosomes
and poor particles
129,369 particles
3D classification
to select RNC
with NAC-NatD
(N of classes: 2;
filter res.: 10 Å;
N of O-EM epochs: 8)
mask for 3D class.
covers NAC-NatD:
3D classification
to select RNC
with NAC-MetAP1
(N of classes: 2;
filter res.: 10 Å;
N of O-EM epochs: 8)
mask for 3D class.
covers NAC-MetAP1:
NatD
NAC
NatD
NAC
MetAP1
RNC-NAC
71,278 particles
RNC-NAC-NatD
78,901 particles
RNC-NAC-MetAP1(-NatD)
73,147 particles
RNC-NAC
77,032 particles
intersect particle sets – 47,409 particles
3D classification with mask on NAC-MetAP1
(N of classes: 4;
filter res.: 8 Å;
N of O-EM epochs: 4)
NatD
MetAP1
NatD
MetAP1
NatD
MetAP1
NatD
MetAP1
poor MetAP1 density
11,784 particles
poor MetAP1 density
11,728 particles
MetAP1 distal conf.
12,691 particles
MetAP1 proximal conf.
11,206 particles
homogeneous refinement
(optimize per-group CTF parameters: true;
optimize per-particle defocus: true)
RNC$_{H4-60aa}$
NAC-MetAP1-NatD
3.55 Å
11,206 particles
EMDB-55351

Fig. S1. Cryo-EM data processing flowchart for the RNC$_{H4\text{-}60aa\text{-}SGRG}$-NAC-MetAP1-NatD dataset. The figure lists data processing steps done in CryoSPARC. Non-default parameters for the jobs are indicated in brackets. Particle stacks selected for further processing steps after 3D classifications are in bold. rRNA is shown in light grey, small subunit proteins in beige, large subunit proteins in light blue, P-site tRNA in red, NACα in yellow, NACβ in orange, MetAP1 in slate blue and NatD in green. Masks used for 3D classifications are shown as semi-transparent green surfaces.

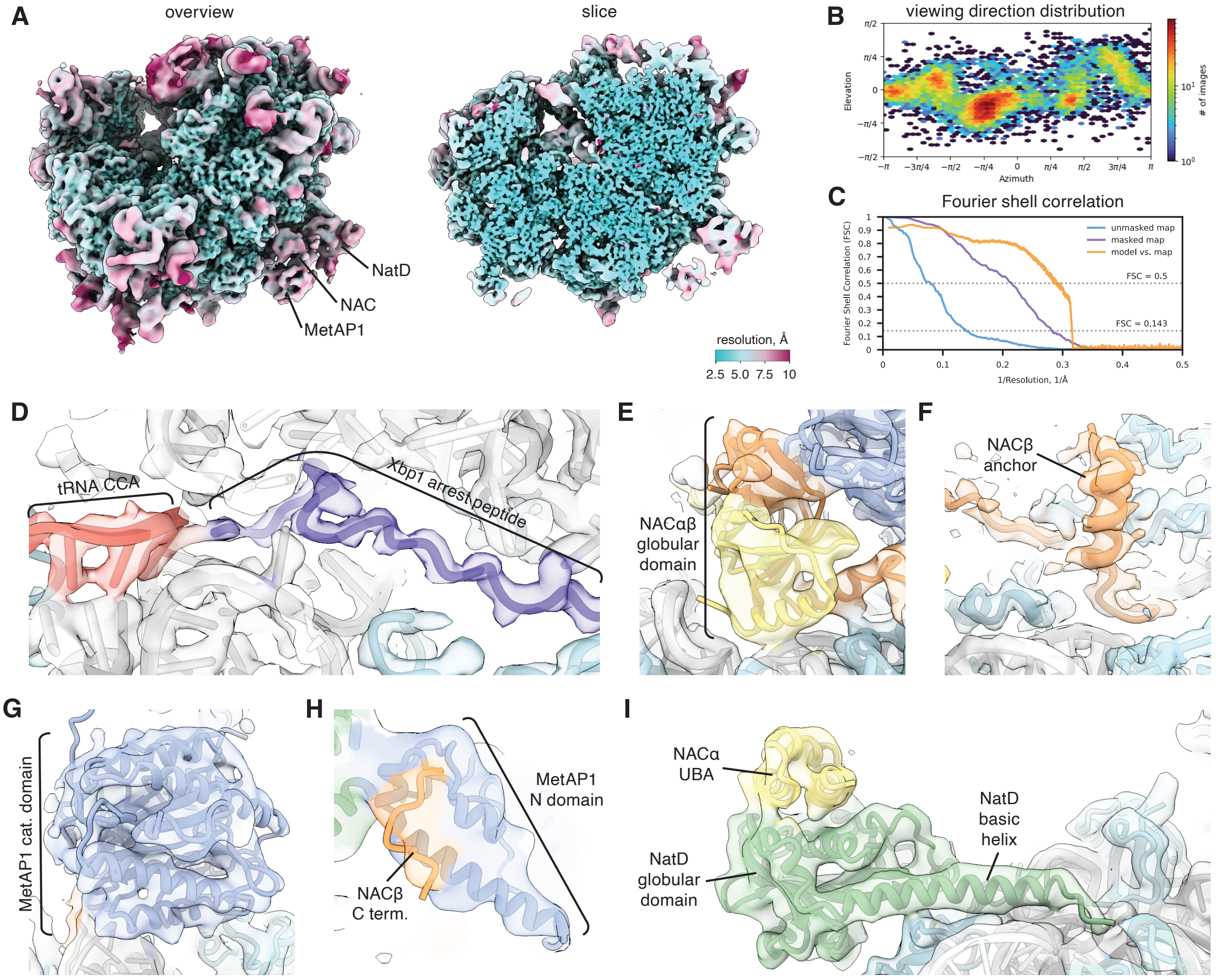

Fig. S2. Local resolution estimates, orientation distribution, refinement statistics and details of cryo-EM maps.

(A) Overview of (left) and a slice-through (right) the cryo-EM map filtered and coloured according to the estimated local resolution in CryoSPARC, coloured on the scale from 2.5 to 10 Å. **(B)** Viewing direction distribution plot heatmap from the homogeneous refinement performed in CryoSPARC. (**C)** Fourier shell correlations (FSC) between unmasked (blue curve) or masked (purple curve) half-maps, and between the map and the model (orange curve). FSC curves for maps and map vs. model were obtained from the outputs of the homogeneous refinement in CryoSPARC and the real-space refinement of the model in Phenix, respectively. **(D-I)** Details of the cryo-EM structure superimposed on the model of the H4 RNC-NAC-MetAP1-NatD complex. The rRNA is shown in light grey, large subunit proteins in light blue, the P-site tRNA in red, the nascent chain in purple, NACα in yellow, NACβ in orange, MetAP1 in slate blue and NatD in green. (D) Detail of the CCA 3’ end of the P-site tRNA linked to the Xbp1 arrest peptide in the ribosomal tunnel. (E) Detail of the globular domain of NAC bound at the ribosomal tunnel exit. (F) Detail of the N-terminal anchor of NACβ. (G) Detail of the catalytic domain of MetAP1. (H) Detail of the N-terminal zinc finger domain of MetAP1 with the bound portion of

NACβ C terminus. (I) Detail of NatD bound on the surface of the large ribosomal subunit. Panels show the main cryo-EM map (D, F) or maps filtered to 6 Å (E, G, I) or 10 Å (H) resolution.

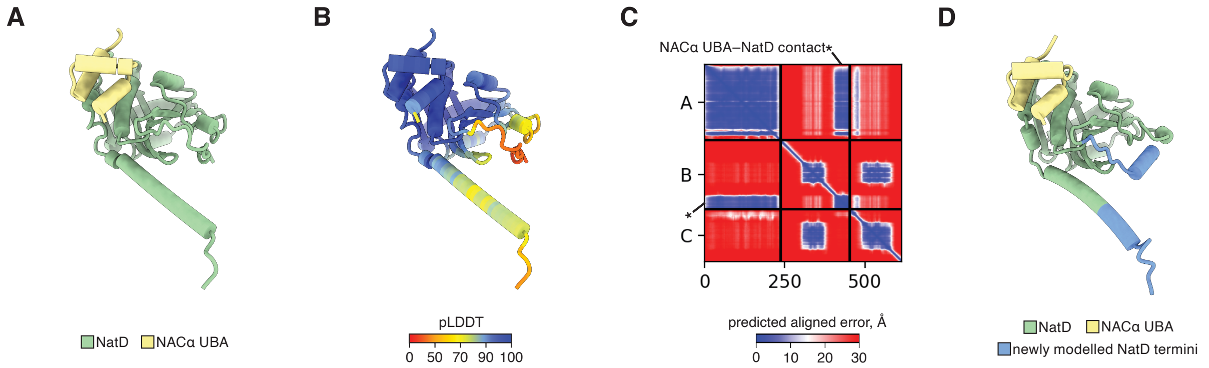

Fig. S3. AlphaFold model of the NatD-NAC complex.

(A, B). Fragment of the AlphaFold-predicted model of NatD-NACα-NACβ complex comprising full-length NatD and the UBA domain of NACα coloured by chain (A) and by pLDDT (B). The displayed fragment matches the cryo-EM map of the RNC-NAC-MetAP1-NatD complex, and the remaining parts of the prediction are not shown for clarity. **(C)** Predicted aligned error (PAE) plot for the AlphaFold-predicted model, with NatD as chain A, NACα as chain B and NACβ as chain C. The chain residues are marked along the x and y axis. The heatmap indicates the estimated position error in Å for residue x when predicted and true structures are aligned on residue y. Low PAE for residue pairs from NatD and NACα UBA (marked on the plot with *) indicate that relative positions of these domains in the AlphaFold prediction are well-defined. **(D).** Fragment of the final RNC-NAC-MetAP1-NatD model comprising full-length NatD and the UBA domain of NACα coloured by chain. NatD termini resolved in our cryo-EM structure but absent in the previously reported crystal structure of truncated NatD (PDB 4U9W) are shown in blue.

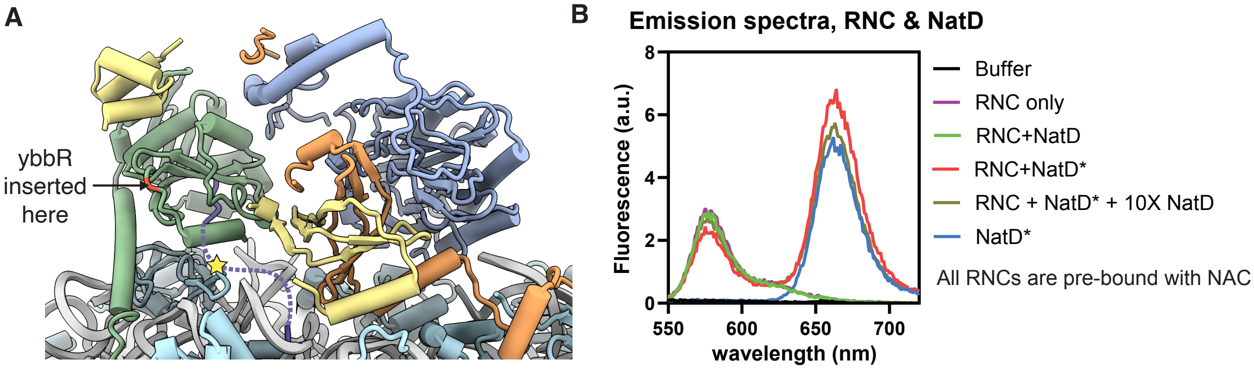

Fig. S4. FRET assay to measure the binding of NatD with RNC_{H4}.

(A) The position of the FRET donor (yellow) and acceptor (red) dyes are depicted in the structural model of the RNC-NAC-MetAP1-NatD complex, with the 60S subunit in light blue and grey, NatD in green, H4 nascent chain in purple, NAC in yellow and orange, MetAP1 in slate blue. **(B)** Fluorescence emission spectra are shown for 2 nM Cy3B labeled $RNC_{H4\text{-}70aa}$ in the absence (purple) and presence of 100 nM unlabeled NatD (green) or Atto647n-labeled NatD (NatD*; red). The RNC + NatD* sample was competed with 1 µM WT unlabeled NatD (brown). NAC (50 nM) is bound to $RNC_{H4\text{-}70aa}$ in all cases. Buffer only (black) and NatD* only (blue) served as references for Raman scatter and contribution of the acceptor dye without donor, respectively. a.u., arbitrary units.

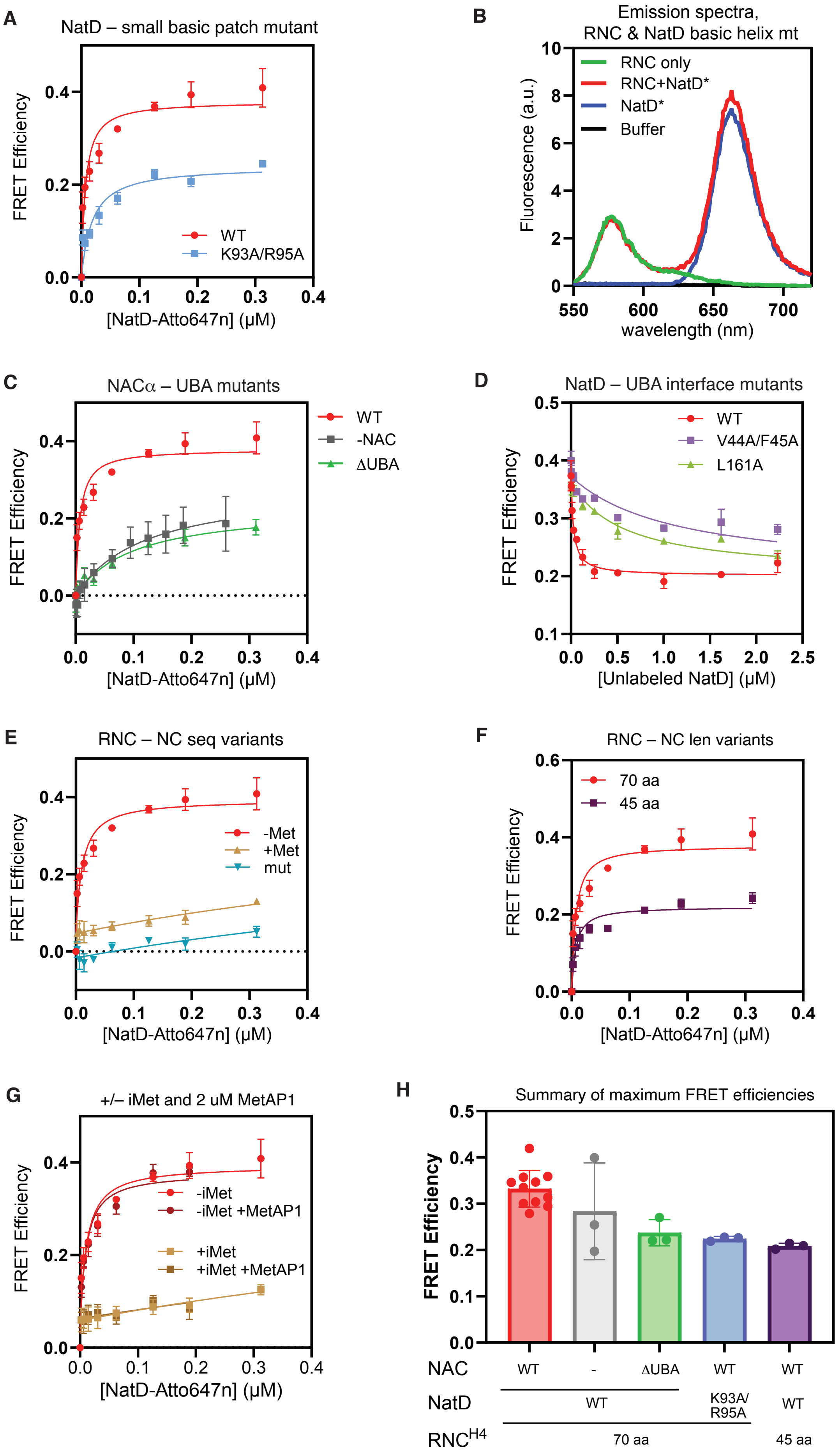
A
NatD – small basic patch mutant
FRET Efficiency
[NatD-Atto647n] (µM)
WT
K93A/R95A
B
Emission spectra,
RNC & NatD basic helix mt
RNC only
RNC+NatD*
NatD*
Buffer
Fluorescence (a.u.)
wavelength (nm)
C
NACα – UBA mutants
WT
-NAC
ΔUBA
FRET Efficiency
[NatD-Atto647n] (µM)
D
NatD – UBA interface mutants
WT
V44A/F45A
L161A
FRET Efficiency
[Unlabeled NatD] (µM)
E
RNC – NC seq variants
-Met
+Met
mut
FRET Efficiency
[NatD-Atto647n] (µM)
F
RNC – NC len variants
70 aa
45 aa
FRET Efficiency
[NatD-Atto647n] (µM)
G
+/– iMet and 2 uM MetAP1
-iMet
-iMet +MetAP1
+iMet
+iMet +MetAP1
FRET Efficiency
[NatD-Atto647n] (µM)
H
Summary of maximum FRET efficiencies
FRET Efficiency
NAC
WT
-
ΔUBA
WT
WT
NatD
WT
K93A/R95A
WT
RNC[H4]
70 aa
45 aa

Fig. S5. FRET-based binding assays with NatD, NAC and RNC variants.

(A) Equilibrium titrations to measure the binding affinity of wild type NatD and mutant NatD-K93A/R95A for NAC-bound $RNC_{H4-70aa}$. **(B)** Fluorescence emission spectra of 2 nM Cy3B-labeled $RNC_{H4-70aa}$ in the absence (green) and presence of 100 nM Atto647N-labeled NatD basic helix mutant (RNC+NatD*; red). NAC (50 nM) is bound to $RNC_{H4-70aa}$ in both cases. Buffer only (black) and NatD* only (blue) served as references for Raman scatter and contribution of the acceptor dye without donor, respectively. a.u., arbitrary units. **(C)** Equilibrium titrations to measure the binding affinity of NatD for $RNC_{H4-70aa}$ bound with wild type NAC and the indicated NAC variants. **(D)** Inhibition assays to measure the binding affinity of wildtype and mutant NatD for $RNC_{H4-70aa}$ bound to wild type NAC (A). Unlabeled NatD is titrated into a preformed complex of Cy3B labeled $RNC_{H4-70aa}$ with Atto647N-labeled NatD. The lines are fits of the data to Eq 3. The obtained inhibition constants (Ki) are summarized in Fig. 3C. **(E)** Equilibrium titrations to measure the binding affinity of NatD for $RNC_{H4-70aa}$ with (+Met) and without (–Met) initiator methionine, and with the N-terminal SGRG sequence of H4 replaced by residues 2-5 from uL4, a NatA substrate (mut). **(F)** Equilibrium titrations to measure the binding affinity of NatD for RNC_{H4} with nascent chain lengths of 70 aa and 45 aa. **(G)** Equilibrium titrations to measure the binding affinity of NatD for $RNC_{H4-70aa}$ with and without initiator methionine in the nascent chain in the presence and absence of 2 μM MetAP1. **(H)** Summary of the maximum FRET efficiency of the fitted curves in all the samples. Values are shown as mean ± s.d., with the dots showing the data from independent titrations. The changes in maximum FRET efficiency likely indicate changes in the NatD orientation relative to the nascent chain. In (A), (C), (E)-(G), the lines are fits of the data to Eq. 2, and the obtained K_d values are shown in Fig. 3. The values are shown as mean ± s.d., with N = 11 for the sample containing WT NatD and NAC, and N = 3 for all other samples.

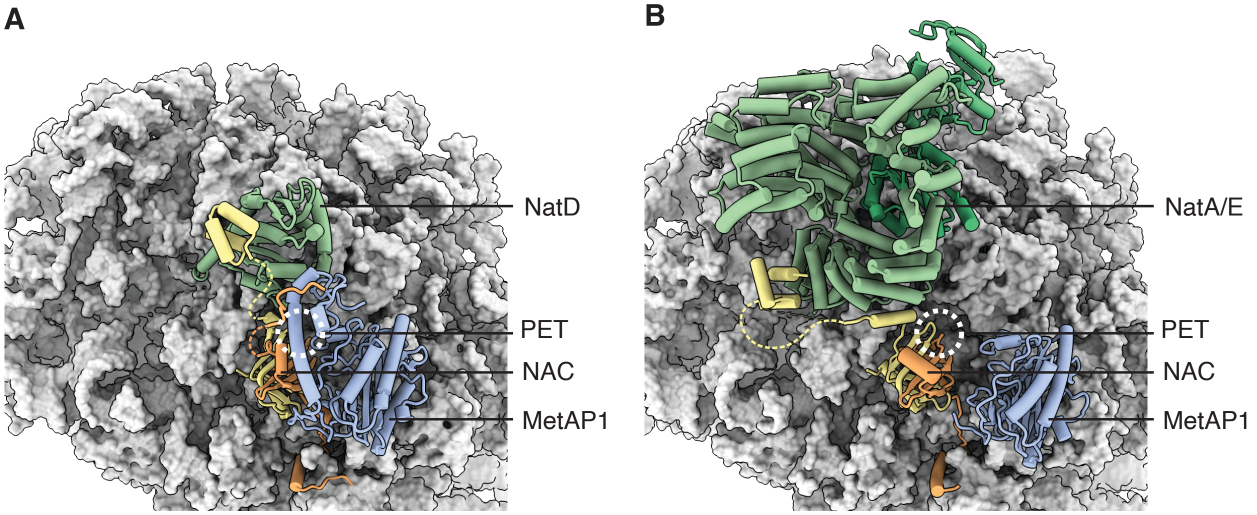

Fig. S6. Binding sites of NatD and NatA/E overlap on the ribosome.

(A) Top view of the RNC_{H4}-NAC-MetAP1-NatD model from this study. (**B)** Top view of the quaternary RNC-NAC-MetAP1-NatA/E complex model from this PDB 9F1C. Models were aligned by the 28S rRNA. Surface of the large ribosomal subunit is shown in grey, NACα in yellow, NACβ in orange, MetAP1 in slate blue and NatD in green, position of the polypeptide exit tunnel (PET) is marked with a white dashed circle.

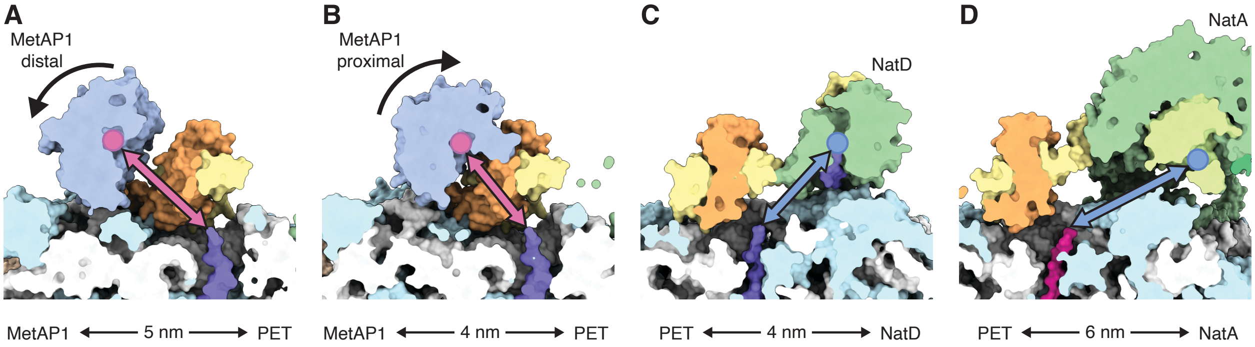

Fig. S7. Distances between PET and active sites of ribosome-bound MetAP1, NatD and NatA.

(A-D) Slices through composite models of RNCs and nascent chain factors with marked distances between PET and the active site of MetAP1 in distal (A; see also Materials and Methods and fig. S1) and proximal (B) conformations, the active site of NatD (C), and the active site of NatA (D). For MetAP1, the crystal structure of the catalytic domain (PDB 2B3H) was docked into respective cryo-EM maps and superimposed on the model of H4 RNC-NAC from this study. The indicated distance is between the N-most residue of the nascent chain modelled in the PET and the chlorine ion bound in the active site of MetAP1. For NatD, the indicated distance is between the N-most residue of the nascent chain visible in PET and the N-most residue of the nascent chain bound by NatD. For NatA, the model of free NatA/E with Ac-CoA bound (PDB 6PPL) was aligned to the model of the quaternary RNC-NAC-MetAP1-NatA/E complex (PDB 9F1C). The indicated distance is between the N-most residue of the nascent chain visible in PET and the acetyl group of Ac-CoA in the substrate cavity of NatA.

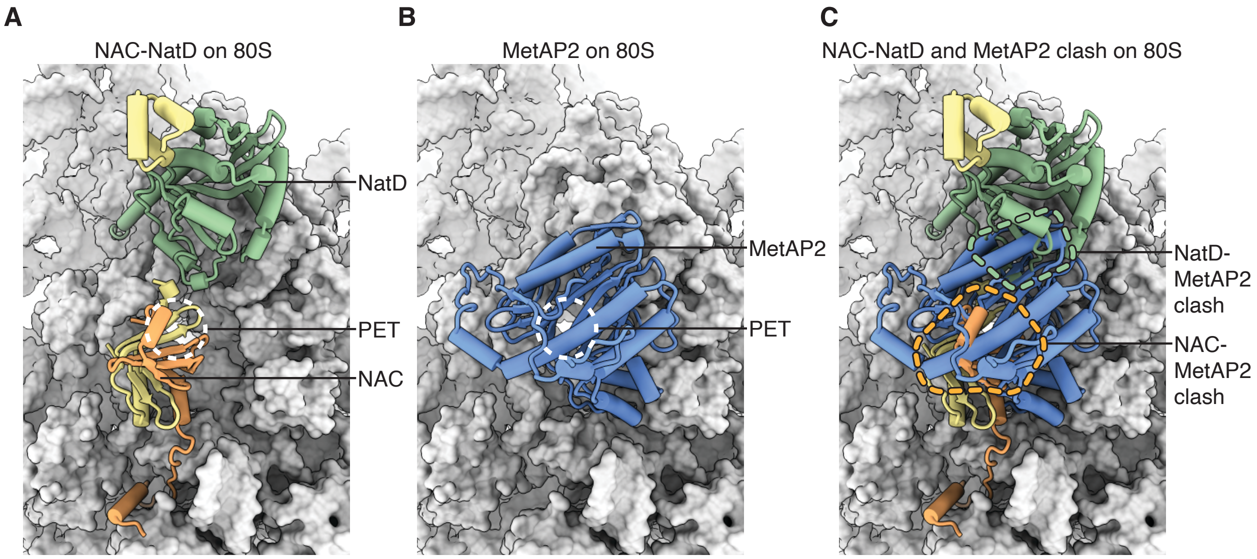

Fig. S8. Comparison between NAC-NatD and MetAP2 footprints on the ribosome.

(A) Position of NAC-NatD on the ribosome. NAC and NatD are shown in cartoon representation, MetAP1 is not shown for clarity, the ribosome is displayed in surface representation. **(B)** Model of MetAP2 from the 80S-MetAP2 complex (PDB 8ONY) displayed over the surface of 80S ribosome model from this study. For superposition, the models were aligned by rRNA. **(C)** Superposition of NAC-NatD and MetAP2 models on the surface of 80S ribosome. Co-binding of NAC-NatD or NatD with MetAP2 on the ribosome is unlikely, as MetAP2 would clash with both NAC and NatD.

Table S1. Cryo-EM data collection, refinement, and validation statistics.

	RNC$_{H4\text{-}60aa}$-NAC-MetAP1-NatD<br>EMD-55351<br>PDB 9SYR
**Data collection and processing**	
Magnification	x81,000
Voltage (kV)	300
Electron exposure (e–/Å^2)	50
Defocus range (μm)	-2.4 to -0.6
Pixel size (Å)	1.065
Symmetry imposed	C1
Initial particle images (no.)	1,282,122
Final particle images (no.)	11,206
Map resolution (Å)	3.55
FSC threshold	0.143
Map resolution range (Å)	2.2 to 10
**Refinement**	
Initial model used (PDB code)	8PPK, 9GMO, 7O7Y, 6R5Q, 7QWR, 2B3H, 4U9W
Model resolution (Å)	3.5
FSC threshold	0.5
Map sharpening B factor (Å^2)	16.1
Model composition	
Non-hydrogen atoms	217,379
Protein residues	12,305
Nucleotides	5,522
B factors (Å^2)	
Protein	59.82/349.13/106.90
Nucleotides	62.89/482.09/107.16
Ligand	90.46/344.60/161.81
R.m.s. deviations	
Bond lengths (Å)	0.003
Bond angles (°)	0.454
Validation	
MolProbity score	1.45
Clashscore	6.09
Poor rotamers (%)	1.00
Ramachandran plot	
Favored (%)	97.39
Allowed (%)	2.58
Disallowed (%)	0.02

Table S2. Summary of the binding affinity of NatD for RNC_{H4}.

Nascent chain	NC len. (aa)	NatD	NAC	MetAP1	K_d (nM) (# replicates)	K_i (nM) (# replicates)	Maximum FRET efficiency (%) (# replicates)
H4-S (^{2}SGRG^5)	70	WT	WT	-	8.6 ± 3.8 (N = 11)	4.3 ± 0.53 (N = 3)	33 ± 3.8 (N = 11)
H4-S (^{2}SGRG^5)	45	WT	WT	-	6.4 ± 1.5 (N = 3)		21 ± 0.51 (N = 3)
H4-S (^{2}SGRG^5)	70	WT	WT	H203A	10.7 ± 4.0 (N = 3)		Set to 33
H4-S (^{2}SGRG^5)	70	WT	-	-	143 ± 78 (N = 3)		28 ± 8.5 (N = 3)
H4-S (^{2}SGRG^5)	70	WT	ΔUBA	-	100 ± 15 (N = 3)		24 ± 2.3 (N = 3)
H4-S (^{2}SGRG^5)	70	V44A/F45A	WT	-		201± 49[a] (N = 2)	N/A
H4-S (^{2}SGRG^5)	70	L161A	WT	-		97± 16[a] (N = 2)	N/A
H4-S (^{2}SGRG^5)	70	Basic helix mt R3A, K4A, K7A, K9A, K11A, K12A, K14A, R15A, R19A	WT	-	N/A[b]		N/A[b]
H4-S (^{2}SGRG^5)	70	K93A/R95A	WT	-	18 ± 4.1 (N=3)		22 ± 0.39 (N = 3)
H4-M (^{1}MSGRG^5)	70	WT	WT	-	2084 ± 705[c] (N = 5)		Set to 33 [c]
H4-M (^{1}MSGRG^5)	70	WT	WT	H203A	1121 ± 389[c] (N = 3)		Set to 33 [c]
H4-mt (^{2}ACAR^5)	70	WT	WT	-	1840 ± 490[c] (N = 3)		Set to 33 [c]

[a] Values are shown as mean ± s.e.m. N, number of technical replicates.
[b] No FRET was detected
[c] Endpoint was set the same as the WT NatD. No significant FRET was observed at the highest protein concentration used. Lower limit of K_d is reported.
[d] The maximum FRET efficiency was set the same as the WT NatD.
